# Impact of African-Specific ACE2 Polymorphisms on Omicron BA.4/5 RBD Binding and Allosteric Communication Within the ACE2–RBD Protein Complex

**DOI:** 10.3390/ijms26031367

**Published:** 2025-02-06

**Authors:** Victor Barozi, Özlem Tastan Bishop

**Affiliations:** 1 Research Unit in Bioinformatics (RUBi), Department of Biochemistry, Microbiology and Bioinformatics, Rhodes University, Makhanda 6139, South Africa; vbarozi2@gmail.com; 2 National Institute for Theoretical and Computational Sciences (NITheCS), Matieland 7602, South Africa

**Keywords:** centrality metrics, dynamic residue network analysis, MD-TASK, MDM-TASK-web, mutation analysis, COVID-19, SARS-CoV-2, vaccine development, personalized treatment

## Abstract

Severe acute respiratory symptom coronavirus 2 (SARS-CoV-2) infection occurs via the attachment of the spike (S) protein’s receptor binding domain (RBD) to human ACE2 (hACE2). Natural polymorphisms in hACE2, particularly at the interface, may alter RBD–hACE2 interactions, potentially affecting viral infectivity across populations. This study identified the effects of six naturally occurring hACE2 polymorphisms with high allele frequency in the African population (S19P, K26R, M82I, K341R, N546D and D597Q) on the interaction with the S protein RBD of the BA.4/5 Omicron sub-lineage through post-molecular dynamics (MD), inter-protein interaction and dynamic residue network (DRN) analyses. Inter-protein interaction analysis suggested that the K26R variation, with the highest interactions, aligns with reports of enhanced RBD binding and increased SARS-CoV-2 susceptibility. Conversely, S19P, showing the fewest interactions and largest inter-protein distances, agrees with studies indicating it hinders RBD binding. The hACE2 M82I substitution destabilized RBD–hACE2 interactions, reducing contact frequency from 92 (WT) to 27. The K341R hACE2 variant, located distally, had allosteric effects that increased RBD–hACE2 contacts compared to WThACE2. This polymorphism has been linked to enhanced affinity for Alpha, Beta and Delta lineages. DRN analyses revealed that hACE2 polymorphisms may alter the interaction networks, especially in key residues involved in enzyme activity and RBD binding. Notably, S19P may weaken hACE2–RBD interactions, while M82I showed reduced centrality of zinc and chloride-coordinating residues, hinting at impaired communication pathways. Overall, our findings show that hACE2 polymorphisms affect S BA.4/5 RBD stability and modulate spike RBD–hACE2 interactions, potentially influencing SARS-CoV-2 infectivity—key insights for vaccine and therapeutic development.

## 1. Introduction

The global pandemic, coronavirus disease 2019 (COVID-19), as declared by the World Health Organization (WHO) [1], is currently (October 2024) responsible for approximately over 770 million cases and over 7 million mortalities [2]. The infectivity of severe acute respiratory syndrome 2 (SARS-CoV-2), a Beta coronavirus responsible for COVID-19 [3,4], is mediated by the human angiotensin-converting enzyme 2 (hACE2) [3,5,6,7]. During infection, the receptor binding domain (RBD) of the SARS-CoV-2 spike (S) protein binds to the peptide domain of hACE2, facilitating viral fusion [8,9].

Human ACE2 is a type 1 membrane carboxypeptidase enzyme and is homologous to the human angiotensin-converting enzyme 1 (hACE1) based on the sequence identity of their extracellular catalytic domain [10]. Carboxypeptidases are involved in the post-translational modification of peptides/proteins through hydrolysis of peptide bonds at the C-terminal end [11]. In humans, ACE2 cleaves Angiotensin (Ang) II to Ang 1–7, which physiological role is vasodilation, anti-inflammatory and anti-proliferative activity, especially in the heart and kidney tissues [12,13]. Consequently, hACE2 activity is linked to cardiovascular function and hypertension [14]. Another role of hACE2 enzyme involves the removal of the leucine residue from the C-terminal end of Ang I, converting it to Ang 1–9 [13]. The enzyme is expressed in various organs and tissues; thus, SARS-CoV-2 can attack many organs with high hACE2 expression [15].

**Figure 1:**
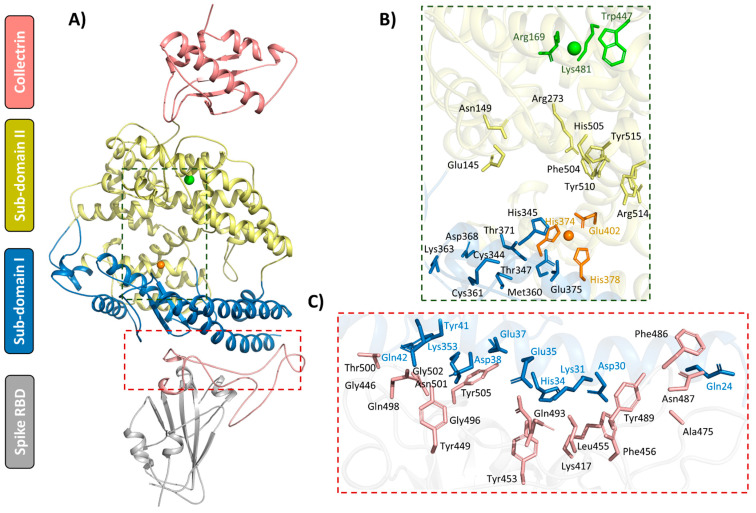
Structural representation of the hACE2 (PDB ID: 7L7F [38]) protein in a complex with the S protein RBD (PDB ID: 6M0J [18]). (**A**) Cartoon representation of the hACE2 sub-domains: the collectrin domain (brown) and the peptidase sub-domains (sub-domain I: blue and II: yellow) in complex with S RBD (grey). The RBD receptor binding motif (RBM) is shown in light brown. (**B**) A zoomed in view of the hACE2 active site pocket showing zinc (orange) and chloride (green) ions. The ion-coordinating residues are shown as sticks in yellow and orange, respectively. (**C**) A detailed view of the interface between hACE2 and S RBD, with the interacting interface residues shown as sticks (blue for hACE2 and brown for RBD).

hACE2 consists of a membrane-bound region and an extracellular/peptidase domain. The extracellular domain is further divided into two distinct regions: the metallopeptidase domain (residues 19–611), which coordinates a zinc ion, and the collectrin-like domain (residues 612–740), sharing over 40% sequence identity with human collectrin [10]. The metallopeptidase peptide domain is further divided into two: a zinc-containing sub-domain I composed of residues 19–102, 290–397 and 417–430 and sub-domain II with residues 103–289, 398–416 and 431–615 [10]. The intersection of the two sub-domains creates a groove across the peptide domain harboring the active site that consists of residues Phe274, Leu278, His345, Asp368, Thr371, Glu375, His378, Glu402, His505, Tyr510, Arg514 and Tyr515 (Figure 1). Interestingly, substrate binding to the active site cleft triggers a closing scissor-like movement of sub-domain I towards sub-domain II, enclosing the substrate in the active site cleft [10]. Furthermore, Lu et al. showed that the binding of SARS-CoV-2 S RBD induced sub-domain II movement towards sub-domain I [16].

The enzymatic function of hACE2 relies on a zinc co-factor coordinated by the conserved HEXXH + E motif, comprising residues His374, His378 and Glu402 and a water molecule [10,11,13]. The enzyme also contains a chloride ion, located distal to the zinc ion and coordinated by Arg169, Trp477 and Lys481, which plays a role in anion regulation [11,17]. Additionally, sub-domain I includes the N-terminal helix (residues 22–57), which interacts extensively with the SARS-CoV-2 S protein through the RBD [18].

Since the emergence of SARS-CoV-2, the virus has undergone numerous genomic alterations leading to the development of variants of concern (VOCs), such as Alpha (B.1.1.7), Beta (B.1.351), Gamma (P.1), Delta (B.1.617.2) and Omicron (B.1.1.529) [19,20,21,22]. These variants are characterized by diverse mutations distributed across the entire SARS-CoV-2 S protein.

The Omicron variant, first documented in South Africa [23], has been the most predominant SARS-CoV-2 VOC for nearly two years [24]. The variant has evolutionally progressed over time, developing new sub-lineages with unique mutations in the S protein RBD, i.e., BA.1, BA.2, BA.3 and BA.4.5 sub-lineages [25,26,27,28].

In our previous study [29], we investigated the evolutionary progression of Omicron sub-lineages BA.1, BA.2, BA.3 and BA.4, focusing on mutations within the S RBD. We demonstrated that the RBD of the BA.4/BA.5 sub-lineages exhibits increased flexibility while forming more interactions with hACE2 compared to the reference Wuhan strain. In addition, the study was the first to identify allosteric communication pathways linking the RBD to the hACE2 core. We revealed, through DRN analysis, that mutations in Omicron sub-lineages induce allosteric alterations in these communication pathways, potentially impacting both the RBD and hACE2 structure and function. Furthermore, other studies have shown that various mutations in the S protein significantly influence the RBD–hACE2 binding affinity and interaction dynamics [30,31,32,33,34].

**Figure 2:**
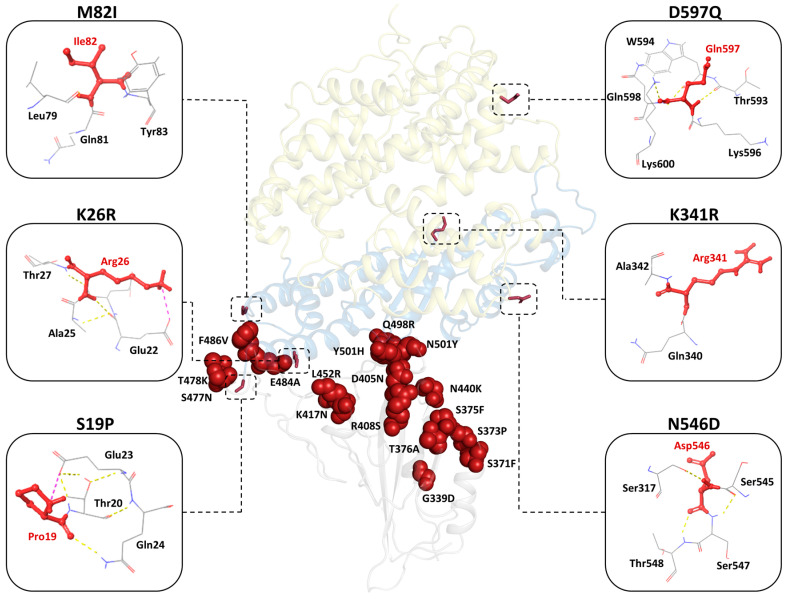
Three-dimensional structural representation of the RBD–hACE2 complex showing the BA.4/BA.5 Omicron RBD mutations (red spheres) and the hACE2 polymorphisms (red sticks). The hACE2 sub-domains I and II are shown in sky blue and pale yellow, respectively, whereas S RBD is in grey. The hACE2 substituted residue interactions within 3 Å are highlighted in yellow, purple and blue for hydrogen bonds (H-bonds), salt bridges and π–π stacking interactions, respectively.

In this paper, we investigate the effects of the hACE2 polymorphisms on the inter-protein interaction between the enzyme and the BA.4/5 Omicron RBD, as well as the changes in allosteric communication patterns. Binding energy studies [35,36,37] have shown that specific hACE2 polymorphisms (K26R, E35D, E35K, T55A, K68E, E75G and T92I), especially at the hACE2–RBD binding interface, influence the protein binding affinity. Biochemical assays conducted by Suryamohan et al. have identified two polymorphic hACE2 proteins, K26R and T92I, as having a higher affinity for the S protein than the reference protein (will also be called wild type; WT) [36]. Conversely, the S protein demonstrated lower affinity for the K31R and E37K hACE2 variants compared to the reference [36]. Building on these findings, this study focuses on six hACE2 polymorphisms, i.e., S19P, K26R, M82I, K341R, N546D and D597Q, that are commonly observed in African populations (Figure 2).

Inter-protein interaction analysis revealed that the hACE2 K26R variation enhances interactions between BA.4/5 Omicron RBD and the human receptor. In contrast, the hACE2 systems bearing the S19P polymorphism exhibited the least number of interactions with the BA.4/5 RBD. The M82I variant was associated with loss of residue centrality in the interface region and an increase in RBD–hACE2 binding energy compared to WThACE2. K341R displayed the highest number of interactions, suggesting that the variation may favor viral binding to hACE2 in the presence of BA.4/5 RBD mutations.

The N546D variation, located at the hACE2 glycosylation site, influenced interactions with the RBD, whereas D597Q exhibited an interaction profile similar to WThACE2. DRN analysis highlighted the allosteric effects of the hACE2 variation on the distribution of high centrality residues in both the hACE2 and RBD systems, impacting the overall protein functionality. The *EC* results further underscored the effect of hACE2 polymorphism on the carboxypeptidase centrality distribution, emphasizing their role in modulating the protein dynamics and interactions.

## 2. Results and Discussion

### 2.1. Selection of High-Frequency hACE2 Polymorphisms in the African Population

The six hACE2 polymorphisms of interest (S19P, K26R, M82I, K341R, N546D and D597Q) were retrieved from the Genome Aggregation Database (gnomAD) [39] using “ACE2” as the search key and selecting missense polymorphisms in African populations with an allele frequency of ≥1.24 × 10^−5^ (Table S1). Allele frequency is the proportion of a gene variant in a given population, and in this case, an allele frequency of ≥1.24 × 10^−5^ corresponds to an allele count of ≥15 for a given variant in a sampled population.

Of these variants, S19P, K26R and M82I are positioned at the interface of the hACE2 N-terminal helix and are involved in the interaction with S RBD [18]. K341R is located in the sub-domain I loop region, whereas N546D and D597Q are in sub-domain II (Figure 2). Position 546 is also identified as a hACE2 glycosylation site [18]. All the variations under study involved residue substitutions of similar physicochemical properties with the corresponding WThACE2, indicating the preservation of structural properties. The 17 RBD mutations of the BA.4/BA.5 Omicron sub-lineage were retrieved from the Global Initiative on Sharing Avian Influenza Data (GISAID) [40], selecting sequences with high sequence coverage, complete and with patient status. CoVsurver [41], a GISAID in-house tool, was used to identify the RBD mutations using hCoV-19/Wuhan/WIV04/2019 (GISAID ID: EPI_ISL_402124) as the reference sequence.

Seven protein complex systems were prepared for this study as detailed in the Methodology section, and these included BA.4/BA.5 RBD–WT hACE2 (WThACE2) and BA.4/BA.5 RBD complexed with each of the six hACE2 polymorphic proteins, namely BA.4/BA.5 RBD–S19P hACE2 (S19P), BA.4/BA.5 RBD–K26R hACE2 (K26R), BA.4/BA.5 RBD–M82I hACE2 (M82I), BA.4/BA.5 RBD–K341R hACE2 (K341R), BA.4/BA.5 RBD–N546D hACE2 (N546D) and BA.4/BA.5 RBD–D597Q hACE2 (D597Q).

Please note that, when it is clear from the context, these complexes, and the individual RBD and hACE2 protein domains of each complex, will be referred to by the relevant polymorphisms as abbreviated above (i.e., WThACE2, S19P, K26R, M82I, K341R, N546D and D597Q).

### 2.2. Effect of hACE2 Polymorphisms on the Global RBD–hACE2 Complex Dynamics

The reference protein complex (WThACE2) and the BA.4/BA.5 RBD complexed with each of the six variant hACE2 proteins were subjected to 400 ns all-atom MD simulations. The computed trajectories were then analyzed by root mean square deviation (RMSD) to evaluate the stability in conformational evolution, root mean square fluctuation (RMSF) for the degree of individual residue flexibility and radius of gyration (Rg) to determine the extent of compaction over the course of the simulations.

**Figure 3:**
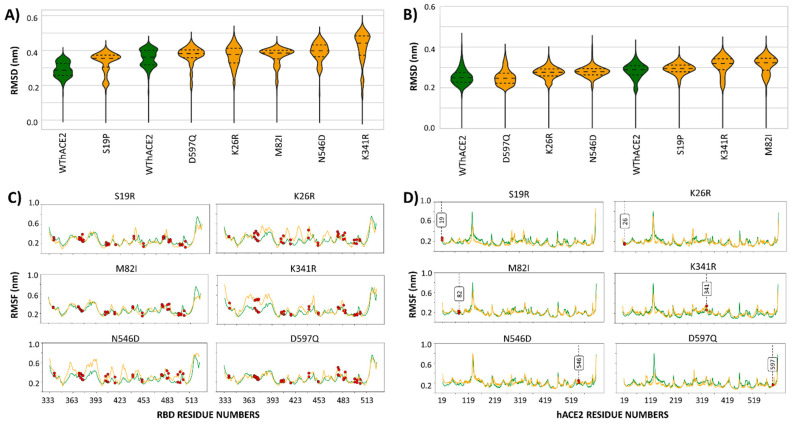
(**A**,**B**) Violin plots showing the RMSD distribution for the RBD and hACE2, respectively, in the WThACE2 and hACE2 variant systems. The plots are arranged in ascending order of the median RMSD, obtained by comparing median values of each system RMSD value. WThACE2 is shown as green and the hACE2 variants in orange. (**C**,**D**) The RMSF for the RBD and hACE2 protein systems, respectively. The same color scheme is used as in (**A**,**B**). Mutation positions in both the RBD and hACE2 proteins are indicated by red markers.

RMSD analysis revealed early MD equilibration for both the WThACE2 and the six hACE2 variant protein complexes. Molecular dynamics reproducibility was ensured through repeated WThACE2 runs, which had an RMSD of less than 0.1 Å between them. Based on this, the observed changes in dynamics in the hACE2 variant systems were attributed to hACE2 variations (Figure S1A,B and Figure 3A,C).

The “RDB-only” trajectory analysis within the protein complex systems showed that the RMSD distributions within the duplicate MD run of the reference system are stable and consistent, with at least two main conformations of close architecture based on the proximity of the violin plot distribution (Figure 3A). However, the RBDs of hACE2 variant systems had one main conformation and smaller spread out RMSD populations in S19P, M82I, K341R, N546D and D597Q (Figure 3A). K341R also featured the highest RMSD distribution. RMSF calculations showed trivial differences in RBD residue flexibility between the WThACE2 and hACE2 variant systems (Figure 3C).

From a hACE2 perspective, both the WThACE2 and the hACE2 variant systems showed more stable RMSD distributions with one main sampled conformation compared to the RBD. Here, the duplicate WThACE2 runs had an average standard deviation of 0.027 between all MD frames, implying no significant differences in RMSD distribution. The M82I hACE2 system experienced the highest structural deviation as per the median RMSD (Figure 3B). The RMSF results also showed trivial variation in residue fluctuations between the hACE2 variant systems and WThACE2 (Figure 3D).

Rg analysis showed nominal margins of change between the reference and the hACE2 variant systems in both the RBD and hACE2 proteins (Figure S1C,D). Taken together, RMSD analysis highlights changes in the RBD protein dynamics consequent to the introduction of hACE2 polymorphisms. The hACE2 variations seem to stabilize the RBD dynamics, which suggests that these hACE2 polymorphisms, with similar phytochemical properties as the WT, have minimal effects on hACE2 behavior, yet they potentially, through allosteric mechanism, influence the RBD dynamics.

Inter-protein interactions are critical in cell regulation and signaling; therefore, sequence changes, for example, through single-nucleotide polymorphisms (SNPs), potentially have a knock-on effect on the interacting protein [42]. This was illustrated in our previous work, where the dynamics of the RBD in Omicron sub-lineages affected the complexed hACE2 conformational distribution [29]. The residue level effects of the hACE2 polymorphisms on the structural networks are further investigated through network analysis in the next section.

### 2.3. Dynamic Residue Networks of the RBD–hACE2 Systems—Local and Global Metric Analysis

In the previous study, we showed that there are well-established allosteric communication paths, defined by residue hubs of DRN metrics, between the Omicron RBD and hACE2 proteins [29]. In the same study, we also demonstrated that the progression of mutations in the Omicron sub-lineages modified these residue networks, and they collectively changed these allosteric paths. Likewise, mutations in the hACE2 protein have the potential to affect both the RBD and hACE2 behavior, as we have already shown from the global protein dynamics in the previous sections. Here, further investigation of possible residue network changes consequent to the hACE2 mutations was done using DRN analysis. This analysis technique has previously identified important and influential residues in different protein systems [43,44,45,46] and highlighted the allosteric communication paths [29,47]. We also discussed the approach in a previous review article [48] and recently applied it to mutation prediction in the SARS-CoV-2 M^pro^ protein, in combination with machine learning approaches [49].

Two slightly different DRN metric data analysis approaches were pursued to calculate residue values for *betweenness centrality* (*BC*), *closeness centrality* (*CC*), *degree centrality (DC)* and *eigenvector centrality* (*EC*) metrics.

(1) Local/system-specific approach: DRN metric values were calculated for each RBD and hACE2 protein within each individual protein system. The top 4% high centrality residues for RBD and top 5% for hACE2 were identified as hubs for each protein system and selected for further analysis. The hubs were visualized as heatmaps to enable the identification of high centrality residues per metric for each protein complex system.

(2) Global analysis approach: This method identified residue hubs from the entire ensemble of RBD/hACE2 systems. The DRN metric values from all systems were combined, and the top 4% and 5% high centrality residues for the RBD and hACE2 systems, respectively, were selected as hubs. Similar to the local approach, these hubs were visualized as heatmaps to examine centrality patterns across systems. Further details on this approach are provided in the Methodology section and referenced in prior studies [43,44,47,48].

#### 2.3.1. The hACE2 Variants Maintained High Centrality Residue Paths Between the RBD and hACE2

It is generally accepted that, within a protein (or a protein complex), communication goes through the shortest paths; hence, finding the shortest paths and residues forming these paths is highly crucial to pinpoint key residues in the information flow. The *BC* metric determines the usage frequency of a residue in the shortest paths calculated between all possible residue pairs within a given network [50,51]. Thus, our averaged *BC* analysis over the MD simulations identifies the key residues with high *BC* values that are considered as information gatekeepers participating in the communication channels.

Figure S2 displays the residue *BC* distribution for each protein system. From an RBD perspective (Figure S2A), we detected a uniform distribution of centrality values across all the systems, characterized by high centrality “peaks” in particular protein regions. The *BC* standard deviation plots for duplicate WThACE2 runs revealed minimal deviations of less than 0.04 in both the RBD and hACE2 proteins. This indicates that the observed changes in centrality within the hACE2 SNP bearing systems can be attributed to the polymorphisms themselves (Figure S3).

The RBD residues around 394–410, 452–457 and 490–515 characteristically had high *BC* values in all the protein systems. These regions form part of the RBD β3 and β7 strands at the protein core, extending further to the RBM at the protein interface. Unlike the RBM, which is tolerant to mutations and harbors at least seven BA.4/5 Omicron sub-lineage mutations, the β3 and β7 strands of the RBD core do not have any BA.4/5 Omicron mutations [29]. Furthermore, Toelzer et al. [52] showed that the RBD core contains a cryptic binding pocket in the proximity of the β3 and β7 strands that binds linoleic acid, which has a stabilizing effect on the RBD in closed conformation. The protein core, composed of β sheets and α helices is also generally important for maintaining structural stability [53]. The RBD regions with the lowest *BC*, i.e., positions 355–390 and 465–480, were prone to mutations (mutation hot spots), with at least six documented Omicron BA.4/5 RBD mutations, i.e., S371F, S373P, S375F, T376A, S477N and T478K.

A uniform *BC* distribution pattern was noted across all the hACE2 systems, regardless of the presence of polymorphisms. This indicates that the mutations did not drastically change the overall protein *BC* pattern (Figure S2B). Here, the high centrality regions included residues 23–26, 72–135, 177–210, 347–364, 371–423, 436–468 and 509–527. Like in the RBD, the high centrality regions were localized at the α-helical regions of the hACE2 core, encompassing the substrate binding site. The high *BC* of the hACE2 inter-subdomain region is justified based on its importance in substrate binding.

**Figure 4:**
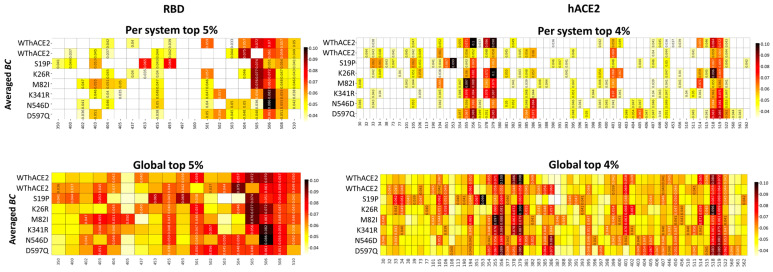
Heatmaps of the top 5% and 4% high *BC* residues in the RBD and hACE2 proteins, respectively, at the individual protein level and global level. Residues are on the x-axis and protein systems on the y-axis. The color scale from white to dark red shows the degree of centrality. The centrality color scale is different for the local and global residues because, in the case of local analysis, centrality calculation is based on the individual systems as opposed to the whole ensemble under the global analysis. The residues with high centrality (hubs) are annotated with metric values in the heatmap, while homologous residues from other systems remain unannotated.

As the next step, we zoomed into residues with high *BC* values, i.e., the top 5% (for RDB) and 4% (for hACE2) high centrality residues as determined by the local/system-specific and the global analysis approach (Figure 4). The two approaches consistently identified the same residues in the majority of cases for both RBD and hACE2 proteins, suggesting that the metric values across individual systems are comparable, with no single protein system exerting dominance over the global analysis.

For most systems, the RBD region spanning residues 500–510 consistently showed high centrality, including the duplicate WThACE2 runs. However, the same region (500–515) in S19P, M82I and K341R had notably low centrality. This region forms part of the β7 strand at the protein core, extending to the interface that stabilizes the RBD. The observed loss of centrality alludes to the reduced protein stability of this region, as discussed in Section 2.2.

Additionally, RBD residues Leu455, Gln506 and Tyr508 had high *BC* values across all the systems, WThACE2 and hACE2 variant systems, at both the protein and global level. As previously observed [29,44,47], the persistence of high centrality across homologous systems (with different residue properties) is an indication of functional importance. Evidently, Leu455, Tyr506 and Tyr508 are positioned at the RBD interface, where they are implicated in forming RBD–hACE2 anchoring interactions [18,54,55,56,57,58]. Tyr506 has also been previously associated with an enhanced binding of SARS-CoV-2 to hACE2 compared to SARS-CoV [59], whereas Tyr508 acts as a binding site for standard drugs, neutralization antibodies, inhibitory peptides and natural inhibitory compounds [60,61,62,63,64]. Additionally, from a global perspective, residue Tyr501 had high centrality in the WThACE2 system and all hACE2 variant systems; however, individual systems analysis showed a significant loss in residue centrality at this position, especially in the S19P system (Figure 4). Tyr501 is known for strengthening RBD–hACE2 interactions [65,66,67], and the loss in centrality possibly highlights the mutational induced effect on Tyr501 involvement in the protein network patterns.

Tyr506 of the N546D system had the highest *BC* value of 0.865 across all the systems, whereas the lowest value, 0.000, was for Gly526 in K341R. Gly526 is in the loop region, where it is not involved in any inter-residue network bridging, which could explain the low centrality.

In the hACE2 protein, we observed a varied distribution of *BC* hubs, with most systems having unique hubs in the presence of hACE2 polymorphisms (Figure 4). However, residues Asp355, Phe356, Ile379, Arg518 and Thr519 consistently maintained high centrality values in all systems (*persistent hubs*: these are residues that maintain high centrality across all the systems) under both individual and global DRN analysis. Asp355 is positioned at the hACE2 interface, where it forms H-bonds across the interface with Thr500 of the RBD. On the other hand, Phe356 is located at the hACE2 cleft, where it interacts with Alacepril [68] and natural flavonoids with potential hACE2-inhibitory properties [69]. Other *persistent hubs*, Ile379 and Arg518, are located at the binding pocket and are implicated in interactions with potential SARS-CoV-2 inhibitors, i.e., verteporfin [70] and isovitexin [71].

From the global analysis, all systems had approximately the same total number of *BC* hubs in the RBD and hACE2 proteins, viz., WThACE2 run 1 (RBD 9 + hACE2 18 = 27 hubs), WThACE2 run 2 (12 + 25 = 37), S19P (11 + 27 = 38), K26R (9 + 23 = 32), M82I (10 + 22 = 32), K341R (9 + 24 = 33), N546D (9 + 26 = 35) and D597Q (12 + 26 = 37).

**Figure 5:**
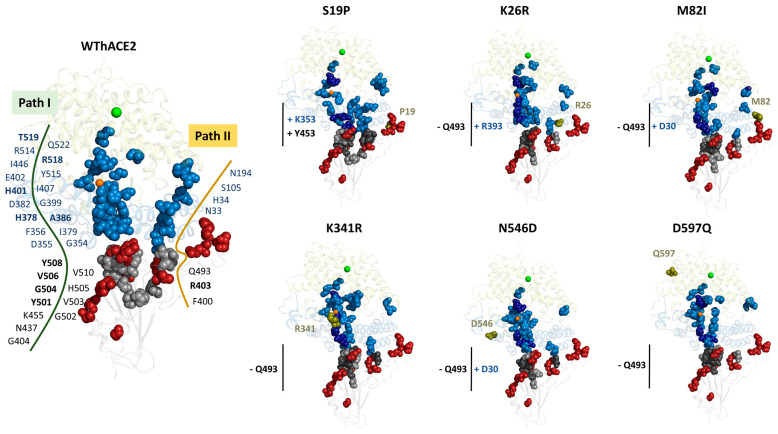
The distribution of the top 5% (RBD) and 4% (hACE2) *BC* hubs in the RBD–hACE2 complexes. For each system, the RBD is shown as a grey cartoon and hACE2 sub-domains I and II as sky blue and pale yellow, respectively. Zinc and chloride ions are shown as orange and green spheres, respectively, whereas the hACE2 mutations are dark green spheres. Hubs (high centrality residues) are shown as sky blue spheres (hACE2) and grey spheres (RBD). The same colors are used for *BC* hubs in the hACE2 variant systems. The five highest centrality *BC* hubs in the RBD and the hACE2 are shown as dark grey and dark blue spheres, respectively. The gains and losses in interface residue hubs in the hACE2 variant systems compared to the WThACE2 are annotated with + and − symbols, respectively. The hACE2 mutation positions are shown as red spheres.

Like previously observed [29], mapped *BC* hubs maintained a network of high centrality residues connecting the RBD to hACE2, irrespective of the presence of hACE2 polymorphisms (Figure 5). In the WThACE2 systems, Path I originated from the RBD core and traversed the receptor binding motif of the S protein and the hACE2 N-terminal domain to the hACE2 active site. Path II connected the RBD core to the other end of the hACE2 opposite to Path I.

In Figure 5, the top five residues with the highest *BC* in the RBD and hACE2 proteins are colored dark grey and dark blue, respectively. Generally, the top five *BC* residues connected the RBD to the hACE2 zinc domain. In the WThACE2 system, the top five RBD residues included Arg403, Tyr501 (*persistent hub*), Gly504, Gln506 (*persistent hub*) and Tyr508 (*persistent hub*) and hACE2 residues, His378, Ala386, His401, Arg518 (*persistent hub*) and Thr519 (*persistent hub*).

For the S19P polymorphism, *BC* analysis showed a gain of *BC* hubs for the interface residue Lys353 in hACE2 and Tyr453 in the RBD compared to WThACE2. In WThACE2, hACE2 Lys353 interacts with Gly496 and Gly502 from the RDB and vice versa at the interface [18]. Furthermore, even though not significant, there was a general reduction in *BC* for the zinc and chloride ion coordinating residues in the S19P complex compared to WThACE2 (Table 1).

Existing research on the effects of hACE2 S19P polymorphism on RBD–hACE2 binding presents conflicting results. Some studies suggest that the S19P polymorphism increases the hACE2 susceptibility to the Wuhan SARS-CoV-2 [36,72]. In contrast, other studies argue that this polymorphism alters the stability and interactions of the hACE2 helix region, leading to reduced receptor affinity for the virus [73,74]. Our analysis suggests that the S19P mutation may, through allosteric mechanisms, negatively impact the S RBD functionality through loss of *BC* for the zinc- and chloride-coordinating residues, as well as reduced centrality for the RBD interface residues 500–510, which play a crucial role in inter-protein interactions.

For the K26R system, previous affinity studies using BLI (bio-layer interferometry) [36] have suggested that the K26R variant has a higher affinity for WT SARS-CoV-2 compared to WThACE2. Here, the K26R system lost *BC* hub status for RBD interface residue Gln493 compared to the WThACE2 system. Structurally, Gln493 forms H-bonds with Glu35 and Glu37 of hACE2 in the RBD–hACE2 complex. Furthermore, K26R also gained an interface *BC* hub, Arg393, in the hACE2 (Figure 5). Unlike S19P, K26R also had more zinc and chloride coordinating residues with higher centrality compared to the WThACE2 system (Table 1).

Based on S19P and K26R systems, it is evident that hACE2 polymorphisms have varied effects on the RBD–hACE2 *BC* centrality, while still maintaining the core communication patterns. Furthermore, the increased centrality of the K26R zinc and substrate binding residues signal to a higher residue activity in the hACE2 K26R system.

Docking studies have previously shown that the M82I hACE2 polymorphism results in lower S RBD binding affinity compared to the WT hACE2. [37]. This is because the mutation is at the protein interface, where it destabilizes the RBD–hACE2 interactions [75]. From *BC* analysis, M82I lost RBD interface residue Gln493 as a *BC* hub and gained Asp30 (hACE2) in comparison to WThACE2. Furthermore, apart from Lys481, all the other important residues in zinc and chloride ion coordination in M82I had lower *BC* values compared to WThACE2. Loss of the *BC* hubs at the interface is possibly a consequence of adjusted interface interactions due to the M82I mutation.

K341R lost Gln493 in the RBD as a *BC* hub (Figure 5). Furthermore, like in K26R and M82I, the top five high centrality hubs in K341R also formed a path connecting the hACE2 interface to the zinc domain. In WThACE2, Gln493 forms interface anchoring H-bonds with Lys31 and Glu35 of hACE2. Therefore, the low *BC* values for the zinc and chloride cofactors emphasizes the effect of the K341R mutation on the enzyme functionality.

N546D gained Asp30 (hACE2) as a *BC* hub but lost Gln493 in the RBD. The loss of *BC* hubs, especially at the RBD Gln493, possibly implies reduced activity of these residues in mediating inter-protein communication along the shortest path. Lastly, D597Q also lost Gln493 as a *BC* hub in the RBD.

The observed gains and losses in hub status are concentrated in the protein interface region, where they influence inter-protein interactions. *BC* analysis revealed subtle shifts in system centrality resulting from hACE2 mutations. These findings suggest that, while hACE2 polymorphisms may alter protein interaction networks, the overall changes are not particularly drastic.

#### 2.3.2. The hACE2 Variants Show Increased *EC* Hubs at the Zinc-Binding Site Compared to WThACE2

In any given network, the influence of a node is based on its degree of connectivity to other highly connected nodes [76]. These influential nodes with high *EC* values influence information dissemination in a network [77]. The MDM-TASK web server [51,78] computes the averaged *EC* of a protein network by averaging the residue network for each frame over the MD simulation.

Unlike *BC*, the RBD *EC* was not uniformly distributed across all the systems. Here, the WThACE2 duplicate runs, K26R and M82I systems had high centrality at positions 493–514, whereas S19P had high centrality around residues 441–450 (Figure S4). The high *EC* “peaks” in K341R, N546D and D597Q were at positions 398–412 and 493–514.

RBD regions 398–412, 441–450 and 493–514 with notably high *EC* values form part of the RBD core extending to the RBM at the interface. Both the protein core and interface are important in protein stability and interaction with hACE2, respectively.

**Figure 6:**
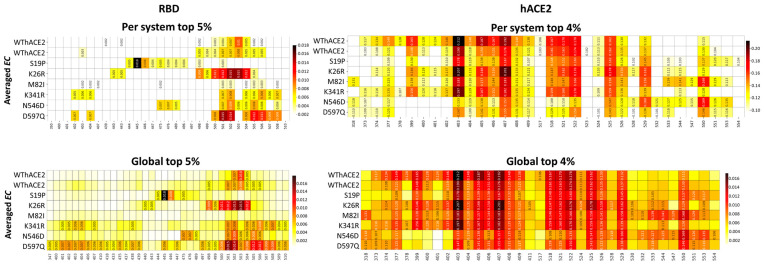
Heatmaps of the top 5% and 4% *EC* residues in the RBD and hACE2, respectively. Residue numbers are on the x-axis and systems on the y-axis. The color scale from white to dark red shows the degree of centrality. The centrality color scale is different for the local and global residues, because in the case of local analysis, centrality calculation is based on the individual systems as opposed to the whole ensemble under the global analysis.

The duplicate WThACE2 runs consistently retained nearly the same *EC* hubs at the local/system-wide top 5% cutoff, suggesting that the shifts in hub distributions observed in the other systems are primarily due to the hACE2 mutations (Figure 6). Here, residue Tyr501 had high centrality across all the systems. The importance of this residue in RBD–hACE2 interactions was earlier highlighted under *BC*. S19P had the highest *EC* (Val445: 0.018) and also harbored unique high centrality residues compared to the other systems, i.e., Lys444-Gly446. These residues form part of the RBM, where Gly446 is known to form H-bonds with Gln42 of hACE2.

Interestingly, no *persistent hubs* were identified for the global RBD *EC* analysis. D597Q had the most hubs, 31, compared to all the other systems (Figure 6). Residues Tyr501-Gly504 had hub status in at least five systems, including the duplicate WThACE2 runs. These residues are positioned at the RBD interface, where they are involved in inter-protein anchoring interactions [65,66,67].

Contrary to the RBD, hACE2 had a uniform *EC* distribution across all the systems, with residues, 368–383, 397–415 and 510–535 having the highest centrality. These residues constitute the α-helical region at the base of the substrate binding cleft, including active site residues Asp368, Thr371, His374, Glu375, His378, Glu402, Tyr510, Arg514 and Tyr515, which are responsible for substrate binding.

In hACE2, per system DRN analysis identified Gly377, Ala403-Ser409, Arg518, Tyr521, Gln522, Phe525, Gln526, Leu529 and Ala550 as high centrality residues based on the top 4% cutoff across all the systems. Similarly, global analysis identified the same residues as *persistent hubs*. These *persistent hubs* also make up part of the α-helix region at the base of the hACE2 active site cleft, where Arg518 has been implicated in coordinating bioactive compounds [79]. Furthermore, Gln522 has previously been shown to interact with Hesperidin, a phytochemical compound from *Saussurea costus*, with SARS-CoV-2-inhibiting potential [80].

**Figure 7:**
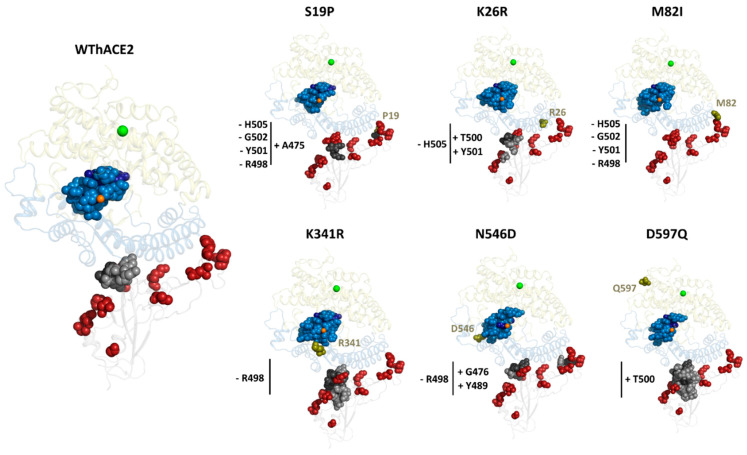
The distribution of the top 5% and 4% RBD and hACE2 *EC* hubs in the RBD–hACE2 complexes. The RBD is shown as a grey cartoon and hACE2 sub-domains I and II as sky blue and pale yellow, respectively. Zinc and chloride ions are shown as orange and green spheres, respectively, whereas the hACE2 mutations are dark green spheres. Hubs are shown as sky blue spheres (hACE2) and grey spheres (RBD). The five highest centrality *EC* hubs in the RBD and the hACE2 are shown as dark grey and dark blue spheres, respectively. The gains and losses in interface residue hubs in the hACE2 variant systems are indicated with + and − symbols, respectively. hACE2 mutations are shown as raspberry spheres.

In general, more *EC* hubs were observed in the hACE2 variant systems compared to the WThACE2 system, viz., WThACE2 run 1 (RBD 3 + hACE2 24 = 27), WThACE2 run 2 (7 + 20 = 27), S19P (4 + 21 = 25), K26R (9 + 28 = 37), M82I (0 + 28 = 28), K341R (15 + 24 = 39), N546D (9 + 25 = 34) and D597Q (31 + 21 = 52). Even though most hACE2 variants generally had more *EC* hubs than WThACE2, the hACE2 interface mutations, i.e., S19P and M82I, showed a significant *EC* hub reduction at the interface compared to the WThACE2 (Figure 7). Based on our understanding of protein residue network patterns, the loss of centrality in these RBM residues likely reflects the negative impact of mutations on inter-protein interactions.

**Figure 8:**
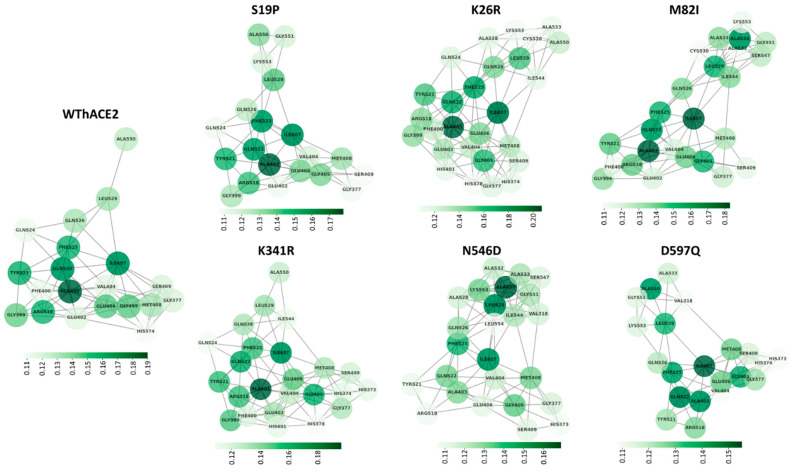
The network of the hACE2 *EC* hubs in each system. The hubs are shown as nodes and their connectedness as edges. The color scale from light to dark green shows the degree of residue *eigenvector centrality (EC)*. The hACE2 SNP bearing systems generally have more connected *EC* hubs, especially involving zinc coordinating residues, signifying the increased influence of this region in protein signal communication.

Furthermore, there was an influx of *EC* hubs at the hACE2 core, including the zinc-binding site residues His374, His378 and Glu402 in the hACE2 variant systems. The *EC* hubs formed a cluster of highly interconnected residues at the base of the substrate binding pocket where the active site and zinc coordinating residues formed a network with the most influential residues in hACE2 (Figure 8). From Figure 8, Ala403, Ile407 and Ala550 were identified as the most influential hACE2 residues. Structurally, Ala403 forms van der Waals interactions with Gln402, which coordinates the zinc ion. In addition, Ile407 forms hydrophobic interactions with Anidulafungin, a fungal drug that has shown inhibitory properties against ACE2–RBD binding [81].

A deeper analysis of the behavior of the hACE2 *EC* hub residues through RMSD and Rg calculations showed a more stable and compact cleft region in the hACE2 variants compared to the WThACE2 (Figure S5A,B). Additionally, there was a correlation between the *EC* hubs and the RMSF. Here, the majority of the *EC* hubs, especially those unique to the hACE2 mutation-containing systems (colored blue in Figure S5C), displayed a reduced degree of residue flexibility compared to their counterparts in the WThACE2 system. A correlation between high *EC* and RMSF has previously been observed [29,44]. Clearly, the hACE2 polymorphisms are associated with a more compact network of the active site and zinc coordinating residues.

The observed differences in network characteristics between WThACE2 and the hACE2 variants show changes in the protein network characteristics around the zinc and active site environment consequent to mutations. These changes to the hACE2 hub network influence hACE2 functionality and RBD binding [35,73,82], which could explain the disparity in SARS-CoV-2 infectivity in different populations.

#### 2.3.3. Distribution of *CC* Values in the RBD–hACE2 Systems

The *CC* values were homogenously distributed in all systems and within a small range of 0.064–0.125 in the RBD and 0.072–0.134 in the hACE2 systems (Figure S6). *CC* informs the connectedness of residues in a network; therefore, a small *CC* range suggests that both the RBD and hACE2 remained stable with a small degree of gyration, as earlier noted in Section 2.2.

The top 5% centrality analysis of the individual RBD systems, Figure S7, identified Arg498, Tyr501 and His505 as high centrality residues across all RBD systems. The RBD Thr500-Gln506 region is a key part of the RBM and plays a crucial role in inter-protein anchoring, as earlier indicated. Thr500 and Tyr501 of the RBD form H-bonds with Tyr41 of hACE2, whereas His505 forms H-bonds with Arg393 of hACE2. Interestingly, only S19P had low centrality for RBD region Tyr500-Gln506 in all the systems.

The RBD global centrality analysis similarly identified Tyr501 as a persistent hub (Figure S7). Among the systems, S19P exhibited the fewest hubs (three), likely due to the loss of interface hubs caused by the hACE2 mutation. Notably, WThACE2 run 2 displayed more *CC* hubs (at positions 403, 447, 449, 455, 493, 494, 495, 496 and 497) compared to run 1. This difference was attributed to variations in the dynamics of the loop regions, where these residues are located.

In hACE2, both local and global analyses identified similar high centrality regions, further highlighting that the network characteristics are consistent across repeated runs. Key residues, including Gly395, Gly399, Phe400, Glu402, Ala403, Val404 and Arg518, emerged as *persistent hubs*. Among these, Gly399, Glu402 and Ala403 also ranked among the top five high centrality residues across the protein systems. These residues are located in the hACE2 cleft, contributing to the substrate binding site. Notably, Glu402 coordinates the zinc ion, which is critical for the enzyme’s carboxypeptidase activity [10]. The functional significance of this region supports the observed centrality of these residues. As with the RBD, the S19P hACE2 system exhibited the fewest hubs compared to other systems. Additionally, the *CC* hubs in hACE2 were predominantly clustered around the active site and zinc-binding region, emphasizing their importance for the enzyme’s function.

**Figure 9:**
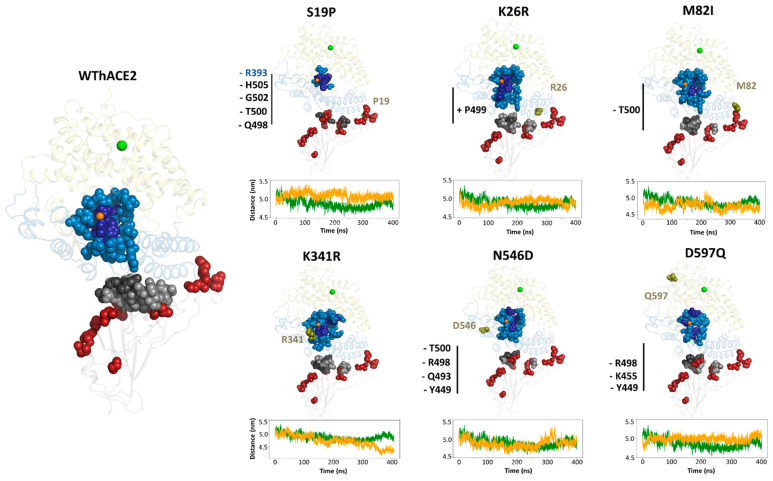
The distribution of the top 5% and 4% *CC* hubs in the RBD and hACE2, respectively. For each system, the RBD is shown as a grey cartoon and hACE2 sub-domains I and II as sky blue and yellow, respectively. The zinc and chloride ions are shown as orange and green spheres, respectively, whereas the hACE2 mutations are dark green spheres. Hubs are shown as sky blue spheres (hACE2) and grey spheres (RBD). The five highest centrality *CC* hubs in the RBD and hACE2 are shown as dark grey and dark blue spheres, respectively. The gains and losses in interface residue hubs in the hACE2 variant systems compared to the WThACE2 are annotated with + and −, respectively. Gained and lost hubs in the hACE2 and RBD variants are annotated in blue and grey, respectively. RBD mutation positions are shown as red spheres. The line subplots in each system show the comparative inter-protein COM distance for the WThACE2 (green) and hACE2 variant systems (orange) over 100 ns.

Compared to WThACE2, the S19P variant lost *CC* hubs at positions Gln498, Thr500, Gly502, His505 and Arg393 (in hACE2). In contrast, the K26R variant showed a gain in *CC* hub status at Pro499, a RBD interface residue known to enhance RBD–hACE2 interactions [83]. In the M82I system, Thr500 lost its hub status compared to WThACE2. For K341R, no changes were observed at the interface; however, N546D lost several key *CC* interface hubs, including Tyr449, Gln493, Arg498 and Thr500 (Figure 9). All the observed gains in *CC* hub status occurred at the interface, underscoring their role in inter-protein communication.

The correlation between *CC* hub distribution at the interface and inter-protein interaction distance is reinforced in this study. S19P, which exhibited the fewest *CC* hubs, also showed the largest inter-protein center-of-mass (COM) distance between the RBD and hACE2 proteins in the complex compared to WThACE2 (Figure 9). This increased interaction distance suggests reduced inter-protein interactions, particularly in the presence of the S19P polymorphism, consistent with the findings by Hussain et al. [73]. The inter-protein interactions are explored in more detail in Section 2.4.

#### 2.3.4. RBD–hACE2 Residue Connectivity as Described by *DC*

*DC* distribution showed a small range between the lowest and highest *DC* values in both the RBD (range: 0.003–0.013) and hACE2 (range: 0.002–0.013) (Figure S8). *DC* analysis across individual systems identified Gly431 and Tyr508 in the RBD as key residues with high centrality. Both residues are located in the RBD core, where Tyr508 plays a crucial role in binding SARS-CoV-2 inhibitors and neutralizing antibodies [61]. Global high centrality analysis revealed minor differences in the RBD centrality distribution between the WThACE2 and hACE2 variant systems (Figure S9). In the RBD, only Tyr501 consistently maintained hub status (*persistent hub*). Tyr501 is crucial for RBD binding, forming H-bonds with Tyr41.

In the hACE2 systems, both local and global analyses identified similar high centrality residues, indicating a uniform centrality distribution across systems. Ala25, Ile88, Val93, Leu97, Ile407, Arg518 and Ala550 were consistently identified as *persistent DC hubs*. In the RBD–hACE2 complex, Ala25 forms hydrophobic interactions with Tyr83 in hACE2, which, in turn, forms H-bonds with Asn487 and Tyr489 of the RBD [18]. Ala25 and Leu97 are also part of hACE2 sub-domain I, whereas Ile407 is part of C-terminus sub-domain II near the zinc ion. Arg518 is at the α-helix region in sub-domain II (Figure S10). The high centrality in these residues is because of their functional role in the protein system.

In summary, both local and global DRN analyses highlight alterations in the interactions of key functional residues known to facilitate hACE2 enzyme activity and hACE2–RBD binding, likely due to hACE2 polymorphisms. The most significant changes were observed in the S19P polymorphism, which may reduce hACE2–RBD interactions.

### 2.4. HACE2 Variants Maintained High RBD–hACE2 Interactions Except S19P

Residue substitutions resulting from SNPs can alter the local protein architecture and residue interactions due to differences in the physicochemical properties and side chains. These structural changes at protein interfaces can modulate protein–protein interactions by either enhancing interactions and reducing the binding energy or weakening interactions and increasing the binding energy.

**Figure 10:**
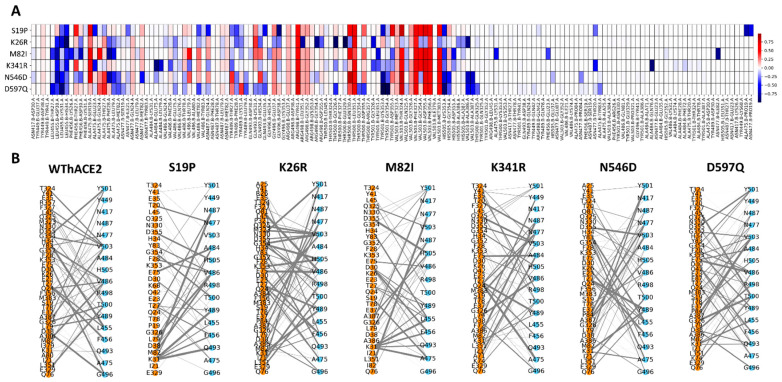
(**A**) The differences between WThACE2 and each hACE2 variant system inter-protein residue interaction frequencies are shown as a heatmap. The differences were calculated by subtracting the variant interface residue pair frequencies from that of WThACE2. The color gradient from red through white to blue shows the delta frequency of residue interactions between the RBD and hACE2. Red means more contact frequency in WthACE2 and vice versa for blue. White shows no difference between the hACE2 variants and WThACE2. (**B**) Bipartite graphs of the weighted pairwise residue interaction frequencies between the RBD residues (sky blue nodes) and hACE2 residues (orange nodes).

We analyzed residue contact maps at the RBD–hACE2 interface using the MDM-TASK web server [51,78] and NetworkX [84] to understand the impact of the hACE2 variations on the RBD–hACE2 interaction in the presence of Omicron BA.4/5 RBD mutations. The differences in residue contact frequency between the WThACE2 and hACE2 variants were computed using delta values defined as the contact frequency of the WThACE2 interface contacts minus that of the hACE2 variant interface residues, as shown in Figure 10A. The bipartite NetworkX plots show the number (edges) and frequency of interactions (edge thickness) between the RBD and hACE2 in each system (Figure 10B).

The hACE2 variant systems generally experienced a significant loss of interactions for several RBD–hACE2 residue pairs compared to WThACE2, including Ala475-Ser19, Arg498-Tyr41, Thr500-Asn330 (except in K26R), Thr500-Leu351, Val503-Thr324 (except in K26R), Val503-Phe327, Val503-Gly354 (except in K26R and N546D), Val503-Phe356 and Val503-Met383 (except in K26R). Except for Ser19, none of the hACE2 polymorphisms studied here are directly involved in these interface interactions, suggesting that the effects observed may be allosterically triggered by the polymorphisms.

In the S19P variant, several residue pairs showed increased inter-protein interactions compared to WThACE2, including Leu455-Lys31, Leu455-His34, Ala475-Glu23, Val486-Met82, Asn487-Gln24, Thr489-Phe28, Gln493-Glu35, Gly496-Asp38, Thr501-Lys353 and His505-Lys353. Conversely, reductions were observed in interactions between Thr27 of hACE2 and Phe456 of the RBD (1.39-fold) and between Asp355 of hACE2 and Tyr501 of the RBD (1.6-fold). Notably, contact map analysis at the hACE2 mutation site (Figure S11) revealed a 1.7-fold and 12-fold increase in the contact frequency of Pro19 with Gly476 and Asn477, respectively (Gly476-Pro19: 0.977, Gly476-Ser19: 0.059 and Asn477-Pro19: 0.485, Asn477-Ser19: 0.04). However, bipartite residue network analysis showed that S19P had the fewest RBD–hACE2 interactions (58) compared to WThACE2 (92).

Overall, we speculate that the S19P polymorphism in hACE2 negatively impacts the BA.4/5 SARS-CoV-2 RBD and hACE2 interactions based on (1) destabilization of the RBD in S19P, resulting in multiple conformations. Such instability can affect the inter-protein interactions, as evidenced by the reduced interaction frequency between the RB and hACE2. (2) The RBD core and interface residues involved in the inter-protein interaction experienced reduced *BC*, *EC* and particularly *CC* hubs in comparison to WThACE2, implying that the communication through these residues was compromised of the S19P variant. (3) The polymorphism also resulted in reduced *BC* at the hACE2 zinc and chloride ions coordinating residues. The ions are vital for the enzyme activity.

The K26R variant presented significantly enhanced RBD–hACE2 interactions compared to WThACE2, with notable gain in the interactions at Lys455-Asp30, Leu455-Lys31, Leu455-His34, Arg498-Leu351, Arg498-Gly354, Thr500-Gly326, Thr500-Glu329, Tyr501-Gly326, His505-Ala386, His505-Ala387 and Val503-Ala386. Additionally, K26R had a total of 87 RBD–hACE2 interactions, slightly fewer than the 92 observed in WThACE2 (Figure 10).

Compared to S19P, the K26R variant exhibited consistently high centrality values for both the RBD and hACE2 interface residues across the DRN metrics. Additionally, the high *EC* values for the RBM in K26R were associated with lower RMSF, indicating more stable complexes. In the native hACE2 structure, Lys26 forms polar interactions with the Asn90 glycan, which can obstruct RBD binding due to steric clashes [85,86]. However, as suggested by Suryamohan et al., the K26R mutation likely disrupts this interaction with the glycan, potentially enhancing RBD binding [36]. The increased *CC* Pro499 in K26R suggests strengthened RBD–hACE2 interactions, in line with the findings from Chunyan et al. [83]. Our analyses of K26R are consistent with previous studies [36,37,74,87,88], which indicates that the K26R polymorphism may favor the interaction between the SARS-CoV-2 RBD and hACE2.

In the M82I complex, changes in RBD–hACE2 interactions compared to WThACE2 were observed at several residue pairs, including Leu455-Thr27, Leu455-Asp30, Leu455-His34, Phe456-Lys26, Ala475-Phe28, Ala475-Leu79, Tyr501-Leu351, Tyr501-Asp355, His505-Lys353, His505-Asp355, Ala475-Lys31, Phe456-Glu23 and Asn477-Ile82. The M82I variant exhibited a total of 71 RBD–hACE2 interactions. At the mutated Met82 position, the substitution resulted in a ~6.6-fold increase in interactions between Ile82 and RBD residue Gly476, as well as a ~2-fold increase with Asn477 (Figure S11). Given that isoleucine and methionine share similar physicochemical properties, the increased inter-protein residue interactions likely stem from conformational dynamics induced by the mutation. Previous studies show that the M82I polymorphism interferes with the RBD–hACE2 interaction in the Wuhan strain. Here, the variant maintained at least 71 residue interactions between the BA.4/5 RBD–hACE2 compared to the 92 in WThACE2, suggesting that the polymorphism has a negative impact on inter-protein interactions.

In the K341R variant, inter-protein interactions shifted in favor of RBD–hACE2 binding compared to WThACE2, with enhanced interactions observed between residue pairs Leu455-Asp30, Leu455-His34, Ala484-Glu75, Ala484-Leu79, Val486-Phe28, Tyr501-Gly326, Tyr501-Leu351, His505-Lys353, His505-Asp355, Asn477-Thr20 and Ala484-Gln76. Notably, the K341R system exhibited the highest number of RBD–hACE2 interactions, totaling 94. Since K341R is distal to the interface region, these effects are likely allosteric. K341R has previously been shown to have increased electrostatic affinity for the Alpha, Beta and Delta variants [89], suggesting that the increased interactions observed here could favor the hACE2 affinity for the Omicron BA4.5 variant.

Similarly, the N546D and D597Q variants exhibited additional interactions at residue pairs Leu455-Asp30, Leu455-Lys31, Leu455-His34, Ala475-Leu79, Ala475-Met82, Tyr501-Lys353, Tyr501-Gly354, His505-Lys353, His505-Gly354, Val503-Ala386 and Val503-Gln325 compared to WThACE2 (Figure 10). The N546D and D597Q systems demonstrated a total of 88 and 92 RBD–hACE2 interactions, respectively. Although the effects of N546D are not well documented, this polymorphism occurs at a glycosylation site [90], which Zhao et al. identified as critical for mediating RBD–hACE2 glycan interactions [91]. The substitution of asparagine with aspartic acid at this position likely disrupts N-linked glycosylation, potentially altering the binding dynamics, as suggested by the observed interaction changes. Alternatively, our analysis indicates that the D597Q polymorphism has an interaction profile nearly identical to that of WThACE2 (92 RBD–hACE2 interactions), suggesting a neutral effect on hACE2 affinity for the RBD, especially in the presence of the BA.4/5 Omicron variant.

Binding energy analysis using the HawkDock [92] webserver revealed that most hACE2 variants had lower RBD–hACE2 binding energy than WThACE2: K26R (−136.69 kcal/mol) > K341R (−82.11) > S19P (−81.79) > WThACE2 (−79.00) > M82I (−78.95) > D597Q (−69.75) > N546D (−69.37). This suggests that the K26R and K341R variations notably enhance binding affinity relative to the other variants.

## 3. Conclusions

The variability in COVID-19 prevalence and severity across different population groups has been partly linked to genetic diversity in ACE2, the human receptor for SARS-CoV-2 [36,93,94,95]. Additionally, variations in hACE2 expression levels are thought to impact SARS-CoV-2 binding and infectivity [96,97].

This paper explored the impact of five naturally occurring hACE2 polymorphisms, i.e., S19P, K26R, M82I, K341R, N546D and D597Q, on the enzyme interaction with the BA.4/5 Omicron sub-lineage RBD. Our findings show that, even though the residue physicochemical properties were conserved throughout the substitutions, the hACE2 variations had both local and allosteric effects on communication patterns and residue interactions between the spike protein RBD and hACE2 receptor.

Mainly, the K26R variation at the interface displayed the highest number of interactions [87], while S19P showed the fewest, consistent with prior research suggesting K26R enhances RBD binding and may increase susceptibility to SARS-CoV-2 infection [36,37,74,87,88]. Furthermore, S19P exhibited the largest inter-protein distances and the fewest interactions, aligning with a body of research suggesting it hinders RBD binding [73,74,98]. Additionally, DRN analysis showed that these hACE2 variations alter communication and network patterns in both hACE2 and S protein RBD. Specifically, *CC* analysis revealed that the S19P polymorphism significantly reduced the number of central hubs at the RBD–hACE2 interface, resulting in fewer inter-protein interactions and increased interaction distance between the RBD and hACE2.

The identification of K26R and S19P mutations in the hACE2 receptor, with distinct effects on S RBD binding, carries significant clinical implications, particularly in the context of SARS-CoV-2 infection and therapeutic development. The K26R variation, which favors enhanced binding of the RBD to hACE2, may contribute to increased viral infectivity, potentially leading to higher transmission rates and more severe disease outcomes. In contrast, the S19P mutation, which impairs S RBD binding, could result in a reduced ability of the virus to enter host cells, potentially offering some level of protection against infection.

The hACE2 M82I interface residue substitution destabilized RBD–hACE2 interactions, resulting in a loss of contact frequency in the variant (27 contact) compared to the 92 in WThACE2. Additionally, DRN analysis, through *BC* and *EC,* showed reduced centrality for the zinc and chloride coordinating residues in the variant, implying reduced participation in the shorted paths of communication. The variant presents an interesting opportunity to explore its potential role in infection control strategies.

The distally located K341R hACE2 variant had allosteric effects on the receptor interactions, resulting in more RBD–hACE2 interface residue contacts (94) compared to the 92 in WThACE2. Previous studies have linked this polymorphism to increased affinity of the hACE2 with Alpha, Beta and Delta VOCs [89].

For the N546D variant, we believe the substitution of ASN with ASP at the hACE2 glycosylation point disrupts N-linked glycosylation, resulting in reduced interactions between the RBD and hACE2 receptor. The D597Q polymorphism showed no significant changes in DRN analysis with WThACE2 and maintained a similar pattern of interface residue contacts as WThACE2.

These changes in the inter-protein dynamics in these variants highlight the importance of understanding ACE2 variants in the context of viral entry and may inform strategies for therapeutic interventions. For example, therapies targeting the Spike–ACE2 interaction, such as monoclonal antibodies or small molecules that block binding, could be tailored to account for the presence of these variations. Additionally, genetic screening for ACE2 variations may aid in predicting individual susceptibility to SARS-CoV-2 infection or disease severity, guiding personalized treatment approaches and public health strategies.

Furthermore, this analysis provides insights into functionally important protein regions and underscores the utility of graph theory and network analysis for studying protein characteristics.

## 4. Materials and Methods

### 4.1. Retrieval and Modeling of the hACE2 and Omicron BA.4/5 RBD Variants

The hACE2 polymorphisms (S19P, K26R, M82I, K341R, N546D and D597Q) were identified from the Genome Aggregation Database (gnomAD) version 2.1.1 [39], which includes data from approximately 730,947 exomes and 76,215 genomes, representing disease-specific and population-wide genetic information from unrelated individuals. Polymorphisms specific to the African population with an allele frequency of ≥1.24 × 10^−5^ were selected from gnomAD. The Omicron BA.4/5 sub-lineage RBD mutations, previously described in Ref. [29], were obtained from GISAID [40], focusing on African sequences with high coverage and available patient data. Notably, the BA.4 and BA.5 Omicron sub-lineages share the same S protein mutations [99,100,101].

In the absence of both variant hACE2 and mutant RBD crystal structures in the RSCB Protein Data Bank (PDB) [102], the reference (WT) RBD–hACE2 structure (PDB ID: 6M0J) [18] was used as a template. First, the BA.4/5 RBD–hACE2 protein system was prepared using PyMOL [103], version 2.5, then each hACE2 mutation introduced to the BA.4 RBD–hACE2 protein complex. The generated variant 3D structures were protonated at the hACE2 physiological functioning pH of 7.0 [12] using the Protein PKa prediction (PROPKA) tool from PDB2QR [104] (version 2.1.1).

### 4.2. All-Atom Molecular Dynamic Simulations

All-atom molecular dynamic (MD) simulations for seven protein complexes (BA.4 RBD–WThACE2 and six BA.4 RBD–hACE2 variant systems) were performed via GROMACS [105] version 2019.4. GROMOS54a7 force fields [106] were used to set up the topology, *top* and *gro* files before placing the systems in a cubic box of 1 nm clearance from the box margins. Prior to minimization, the cubic box was solvated with the single point charge 216 (SPC216) [107] water model and the system charge neutralized using 0.15 M NaCl ions. Minimization was achieved through the steepest descent energy minimization algorithm, where a step size of 0.01 was used and 1000.0 kJ/mol/nm set as the threshold of a minimized system. No constraints were set for minimization. Minimization was succeeded with equilibration, which was performed in two steps: (1) temperature equilibration under the NVT (constant number of particles, volume and temperature) ensemble using Berendsen temperature coupling at 300 K for 100 ps) and (2) pressure equilibration under the NPT (constant number of particles, pressure and temperature) using the Parrinello–Rahman barostat [108] at 1 atm and 300K equally for 100 ps. The equilibrated systems were then subjected to 400 ns MD simulations with a step size of 0.002 femtoseconds at the Center of High-Performance Computing (CHPC). Here, the LINCS algorithm [109] was used for the equilibration and production runs, where all bonds were constrained, whereas Particle Mesh Ewald (PME) electrostatics [110] were used for long-range electrostatic calculations with a Fourier spacing of 0.16 nm. For short-range Coulomb and van der Waals interactions, a cutoff distance of 1.4 nm was used.

The generated trajectories were stripped of periodic boundary conditions and analyzed using the GROMACS inbuilt tools for the RMSD, root mean square fluctuation (RMSF), radius of gyration (Rg) and center-of-mass distance (COM). The inbuilt tools included *gmx rms*, *gmx rmsf*, *gmx gyrate* and *gmx distance*, respectively. The generated data were analyzed and presented using Seaborn [111], Pandas [112], Matplotlib [113], Numpy [114] and pytraj [115] Python packages. Trajectory visualizations were done using the Visual Molecular Dynamic (VMD) tool [116].

### 4.3. Dynamic Residue Network Analysis

To examine the residue-level effects of hACE2 polymorphisms on RBD–hACE2 interactions and network configurations, DRN analysis was performed using the *calc_network.py* tool, https://github.com/RUBi-ZA/MD-TASK/tree/mdm-task-web/src (accessed on 15 October 2024), available from the MDM-TASK web server [51,78]. Four centrality metrics—average *BC*, *CC*, *DC* and *EC*—were computed for each protein system over the entire MD trajectory. In this DRN framework, averaged residue interaction networks from the MD frames are represented with residues as nodes and interactions within a set threshold (6.7 Å) as edges [51,117,118,119].

Each centrality metric provides insights into unique network properties. Specifically, *BC* measures the extent to which a given node lies along the shortest paths between any pair of nodes, highlighting residues that frequently mediate interactions between other residue pairs. The average *BC* is computed as outlined in Equation (1) [78], where *V* is the number of nodes, *m* is the number of frames, *σ*(*s*,*t*) represents the shortest paths between nodes *s* and *t*, *σ*(*s*,*t∣v*) denotes paths passing through node *v* and *i* indexes each frame. Residues with high *BC* values are considered important in controlling the information flow (gatekeepers) in a network.

(1) $$\bar{BC}\left(v\right)=\frac{1}{m}\sum_{i=1}^{m}\sum_{s,t\in V}\;\frac{\delta \left(s_{i},t_{i}|v_{i}\right)}{\delta \left(s_{i},t_{i}\right)}$$

*CC* identifies which nodes on average are closest to all other nodes in a network. These nodes therefore act as fast information dissemination points. The averaged *CC* is computed using Equation (2) [78], where, *d*(*v*, *u*) and *n* are the shortest-path distance between *v* and *u*, whereas *n* is the number of nodes in the graph, respectively.

(2) $$\bar{CC}\left(v\right)=\frac{n-1}{m}\sum_{i=1}^{m}\sum_{u=1}^{n-1}d\left(v,u\right)$$

*DC* quantifies the number of immediate neighboring nodes connected to a given node. Nodes with a high number of connections have high DC values. While *DC* offers insights into the local connectivity, it does not reflect the node’s overall centrality within the entire network [47]. According to *DC* Equation (3) [78], *n* represents the number of nodes, and *Aijk* denotes the *jk*th adjacency for the *i*th frame.

(3) $$\bar{DC}\left(k\right)=\frac{1}{m\left(n-1\right)}\sum_{i=1}^{m}\sum_{j=1,j\neq i}^{n}A_{ijk}$$

*EC* measures how connected a node is and how important those connections are. It starts with *DC* but goes further by giving more weight to connections with highly important nodes. This helps identify key residues that are linked to other influential residues in the network [47]. Consequently, *EC* highlights important residues that are linked to other significant residues within the network.

*EC* is calculated using Equations (4)and (5) [78]. In Equation (4), *EC* represents the eigenvector, and λ is the eigenvalue resulting from the eigen decomposition of the adjacency matrix *A*. In Equation (5), the averaged *EC* for the *i*th residue is determined by computing the eigenvector for each MD frame and averaging the results.

(4) $$A\cdot \vec{EC}=\lambda \cdot \vec{EC}$$

(5) $$\bar{EC}\left(i\right)=\frac{1}{m}\sum_{k=1}^{m}{EC}_{ik}$$

### 4.4. Identification of the Local and Global RBD and hACE2 High Centrality Hubs

The results from the averaged DRN calculations were further analyzed to identify residues with high local and global centralities across all protein systems, commonly referred to as hubs [29,43,44,47,48]. For the local analysis, the DRN results for each individual system were organized in descending order to pinpoint the top 4% of residues for hACE2 and the top 5% for the RBD, utilizing an ad hoc Python script. For the global analysis, metric-specific results from the entire protein ensemble (RBD and hACE2 separately) were sorted to determine the global cutoff values for the top 5% and 4% of residues in the RBD and hACE2 ensembles, respectively. These cutoff percentages were carefully selected based on protein size to minimize noise while remaining informative.

The established cutoffs were then employed to create a binary matrix for the ensemble metric results, with a value of 1 indicating hubs (≥cutoff) and 0 indicating non-hubs (<cutoff). Heatmaps were generated based on this matrix data, where hubs were annotated with their centrality values, while non-hub residues from other systems were not annotated. Additionally, *persistent hubs*—those identified across the protein ensemble—were determined for each DRN metric.

### 4.5. Inter-Protein Residue Contact Analysis

The effects of the hACE2 polymorphisms on RBD–hACE2 interactions were further investigated through contact map analysis of the RBD interface residues involved in these interactions. The RBD interface residues implicated in receptor interactions were identified through a literature search [18,83,120,121,122] and alanine scanning conducted via the ROBETTA web server [123]. The frequency of short-range contacts among RBD interface residues was assessed, focusing on those within a Euclidian distance of 6.7 Å. This analysis was performed using the *contact_map.py* Python script, https://github.com/RUBi-ZA/MD-TASK/tree/mdm-task-web (accessed on 15 October 2024), from the MDM-TASK web server. The difference between each residue pair contact frequency in the WThACE2 and the hACE2 variants was obtained by computing the delta values (WthACE2 residue pair contact frequency—hACE2 residue pair contact frequency). Heatmaps of the delta values were generated using Seaborn [111] to visualize the contact frequency, while NetworkX graphs [84] were employed to represent the system contacts.

## Figures and Tables

**Table 1:** *BC* values for hACE2 zinc and chloride coordinating residues per system. The up and down arrows show the gain and loss in centrality, respectively, compared to the WThACE2 system.

Residue	WThACE2	S19P	K26R	M82I	K341R	N546D	D597Q
Arg169 (Cl)	0.009	0.009	0.010 **↑**	0.008 **↓**	0.010 **↑**	0.014 **↑**	0.014 **↑**
His374 (Zn)	0.020	0.015 **↓**	0.019 **↓**	0.016 **↓**	0.020	0.014 **↓**	0.018 **↓**
His378 (Zn)	0.086	0.032 **↓**	0.072 **↓**	0.063 **↓**	0.078 **↓**	0.034 **↓**	0.051 **↓**
Glu402 (Zn)	0.049	0.025 **↓**	0.067 **↑**	0.021 **↓**	0.041 **↓**	0.027 **↓**	0.029 **↓**
Trp447 (Cl)	0.023	0.018 **↓**	0.019 **↓**	0.017 **↓**	0.021 **↓**	0.019 **↓**	0.018 **↓**
Lys481 (Cl)	0.009	0.012 **↑**	0.014 **↑**	0.015 **↑**	0.014 **↑**	0.010 **↑**	0.013 **↑**

## Data Availability

All data reported in this article are presented in the article and the Supporting Information section. Dynamic residue network analysis metric scripts are implemented in the MDM-TASK web platform (https://mdmtaskweb.rubi.ru.ac.za/, accessed on 15 October 2024) and are available at https://github.com/RUBi-ZA/MD-TASK/tree/mdm-task-web, accessed on 15 October 2024. MD simulations will be made available upon request.

## Supplementary Materials

The following supporting information can be downloaded at: https://www.mdpi.com/article/10.3390/ijms26031367/s1.
