# Supplementary material for: Impact of African-Specific ACE2 Polymorphisms on Omicron BA.4/5 RBD Binding and Allosteric Communication Within the ACE2–RBD Protein Complex
Source: Int J Mol Sci. 2025 Feb 6;26(3):1367. doi: 10.3390/ijms26031367 (PMC11818624; doi:10.3390/ijms26031367)
Supplement: Supplementary file 1 [file ijms-26-01367-s001.zip › ijms-3319438-supplementary.pdf]

Impact of African-Specific ACE2 Polymorphisms on Omicron BA.4/5 RBD Binding and Allosteric Communication Within the ACE2–RBD Protein Complex

Victor Barozi[1], Özlem Tastan Bishop[1, *]

[1]Research Unit in Bioinformatics (RUBi), Department of Biochemistry, Microbiology and Bioinformatics, Rhodes University, Makhanda 6139, South Africa

Table S1: African specific hACE2 polymorphisms with an allele frequency of ≥ 1.24x10-5 as obtained from the Genome Aggregation Database.

**Mutation**	**dbSNP ID**	**Allele count**	**Allele Frequency**
S19P	rs73635825	124	1.03e-4
K26R	rs4646116	6821	5.64e-3
M82I	rs766996587	15	1.24e-5
K341R	rs138390800	271	2.24e-4
N546D	rs761944150	23	1.90e-5
D597Q	rs145437639	49	4.05e-5

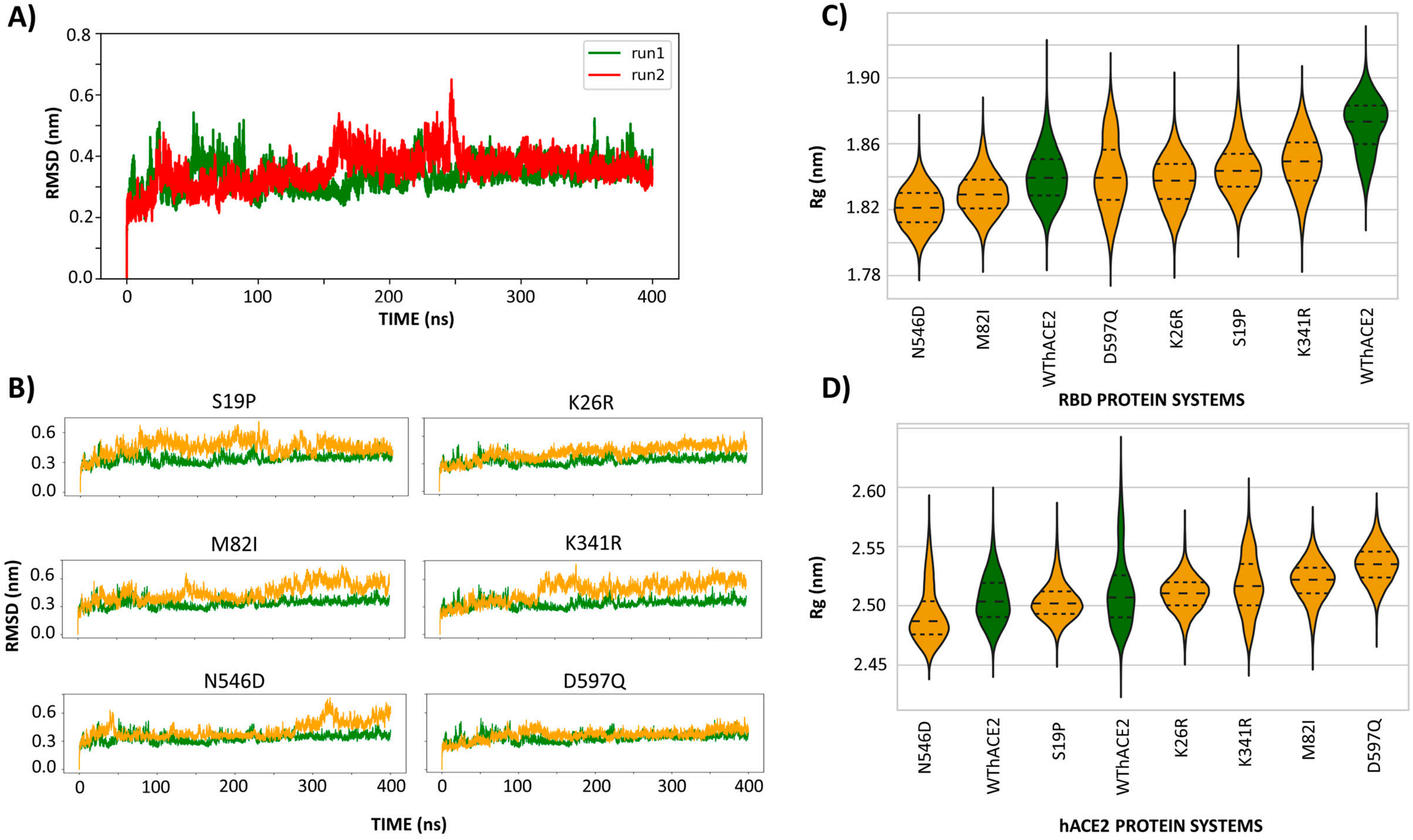

Figure S1: A) and B) are comparative RMSD line plots between the WThACE2 duplicate runs and between the reference (WThACE2) system (green) and the hACE2 variant systems (orange), respectively. The x-axis is time in ns and y-axis the RMSD in nm. C) and D) show the Rg violin plot presentation for the RBD and hACE2 protein systems, respectively. The BA.4 and the hACE2mutation bearing systems are shown in green and orange respectively.

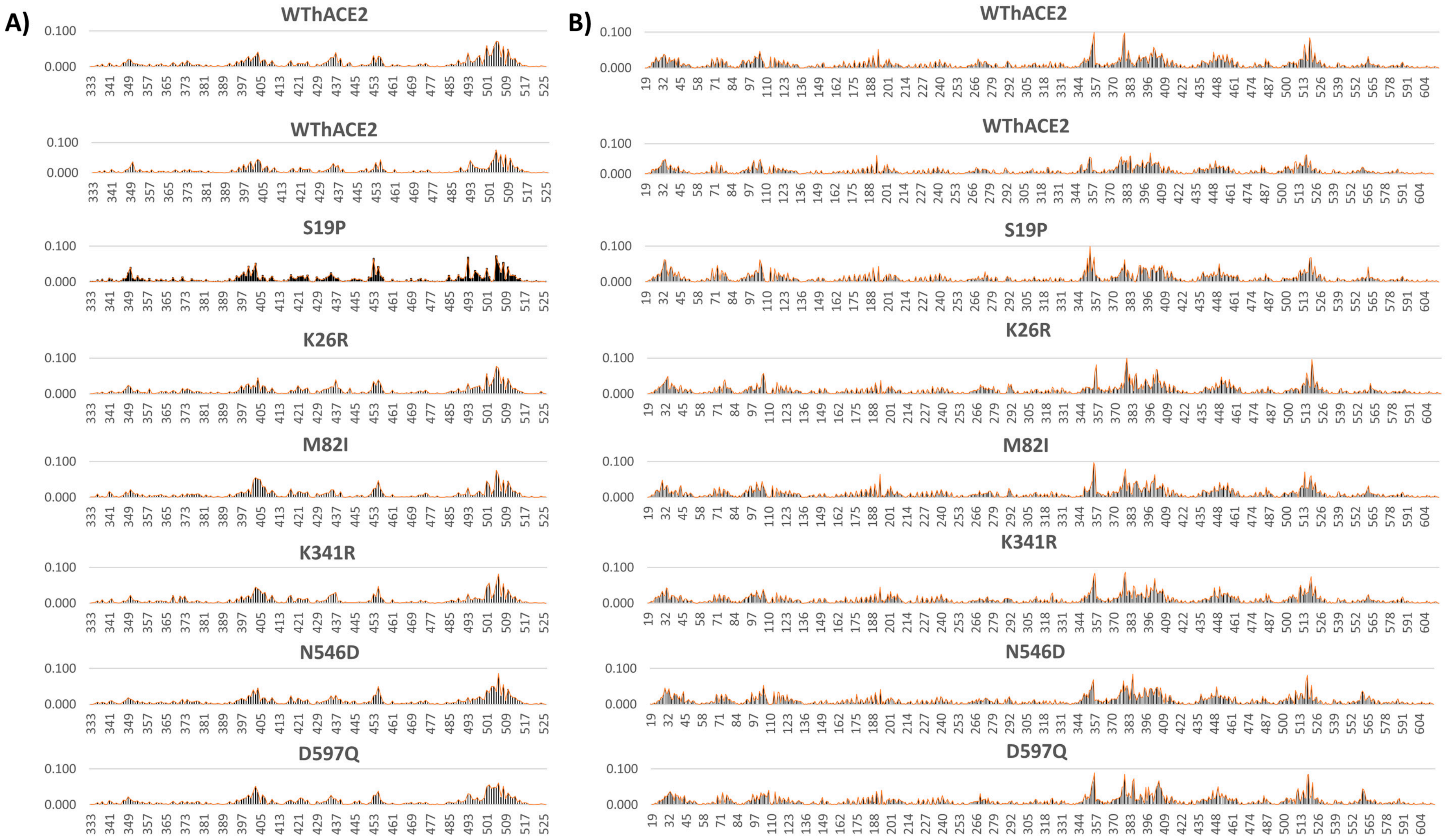

Figure S2: A) and B) show the *BC* distribution in the RBD and hACE2 proteins for each system. The x and y-axes show the residue numbers and the centrality values, respectively. The bar length represents residue centrality.

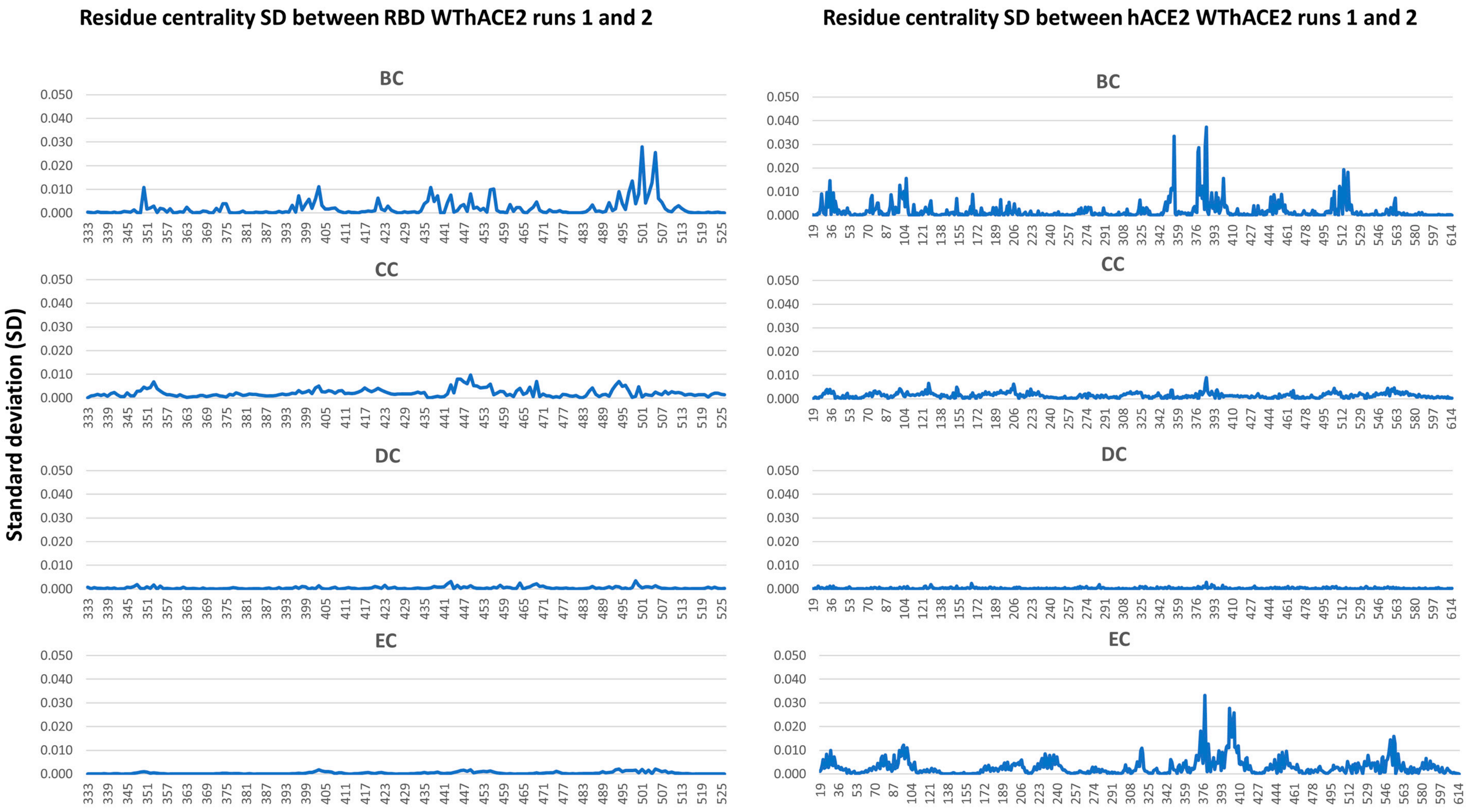

Figure S3: Line plots of the residue standard deviation between the duplicate WThACE2 runs for the RBD and hACE2 proteins. Residue IDs are on the x-axis and SD values on the y-axis.

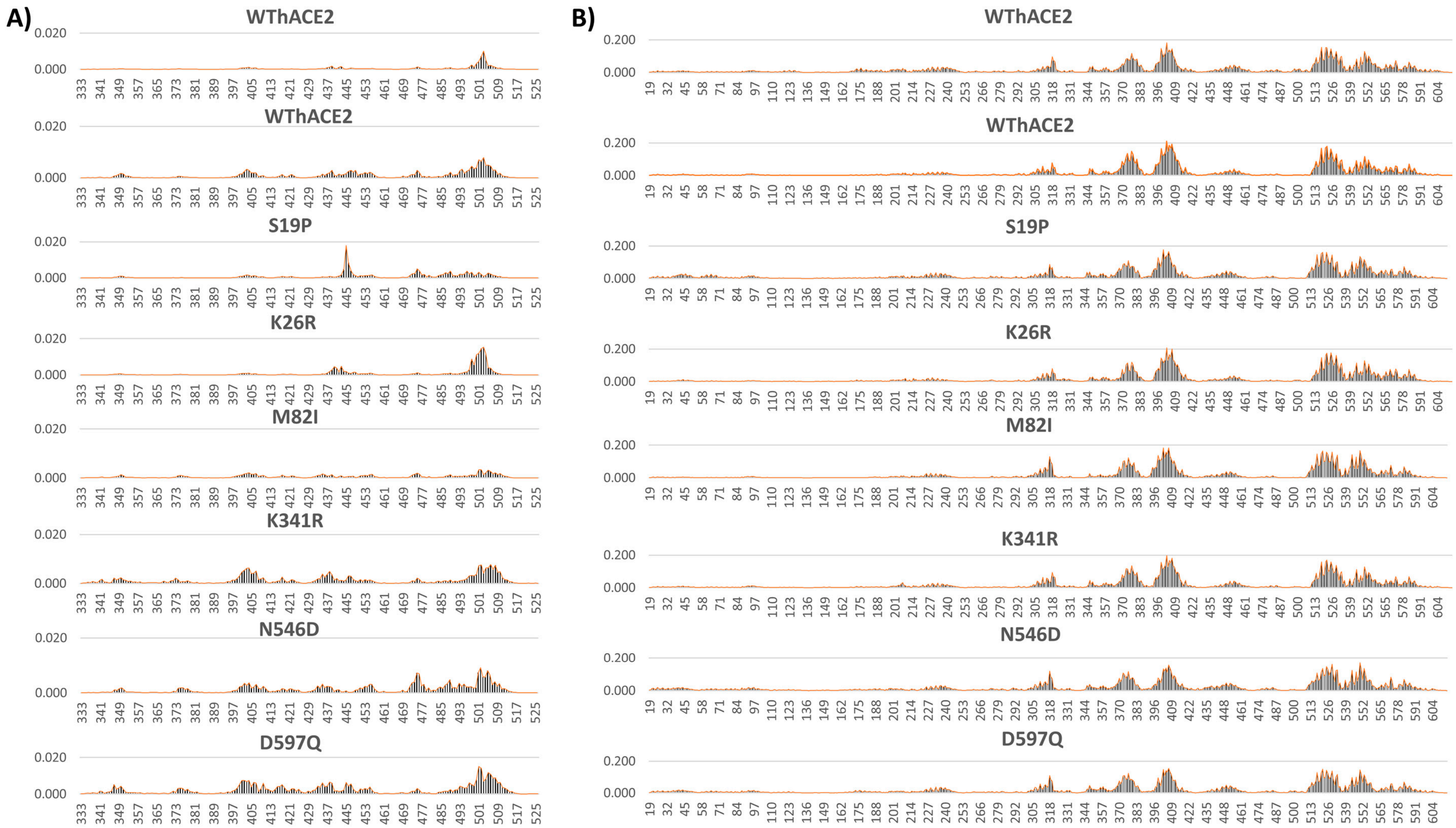

Figure S4: A) and B) show the *EC* distribution in the RBD and hACE2 proteins for each system. The x and y-axes show the residue numbers and the centrality values, respectively.

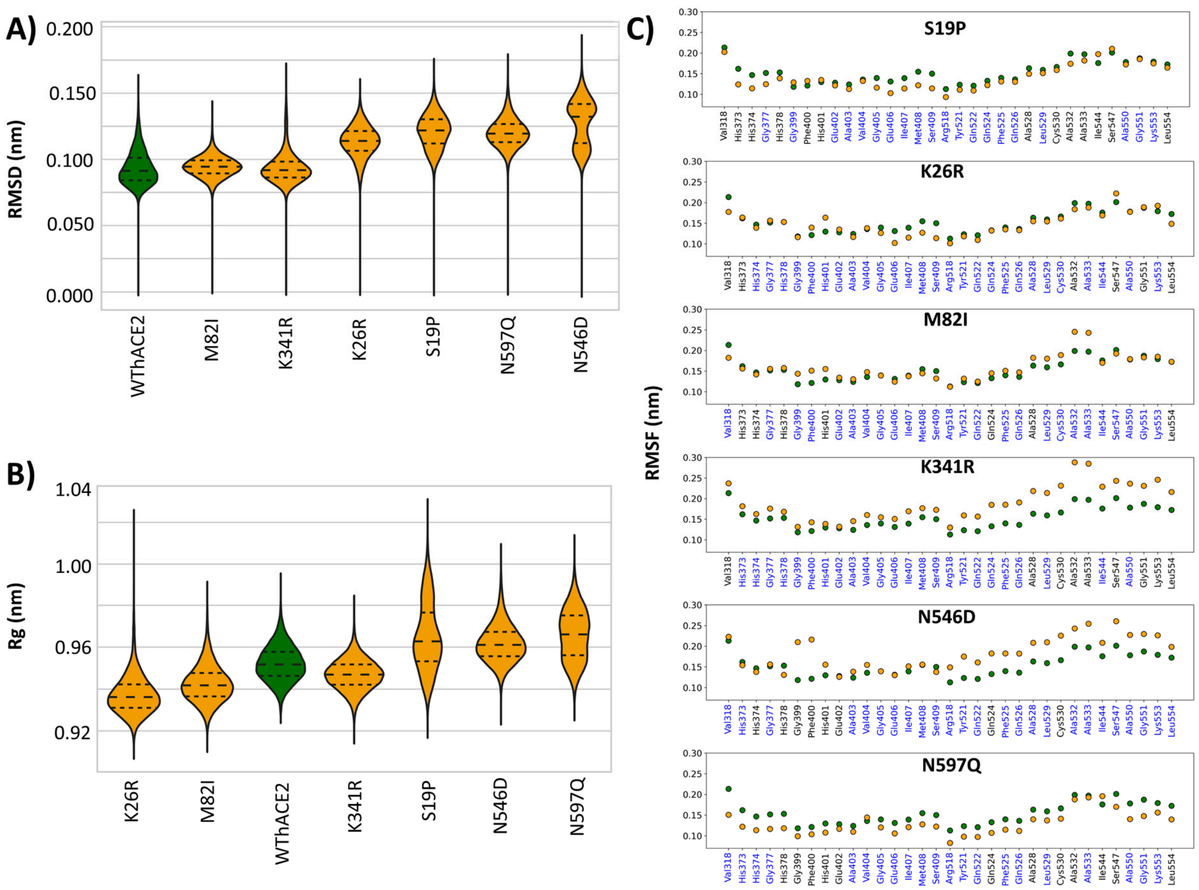

Figure S5: A) and B) are violin plots showing the distribution *EC* hubs RMSD and Rg, respectively, for the WThACE2 (green) and hACE2 variant systems (orange) arranged in ascending order of the median RMSD and Rg. C) Shows the RMSF of the *EC* hub in the hACE2 protein. *EC* hubs unique to the hACE2 variant systems are colored blue.

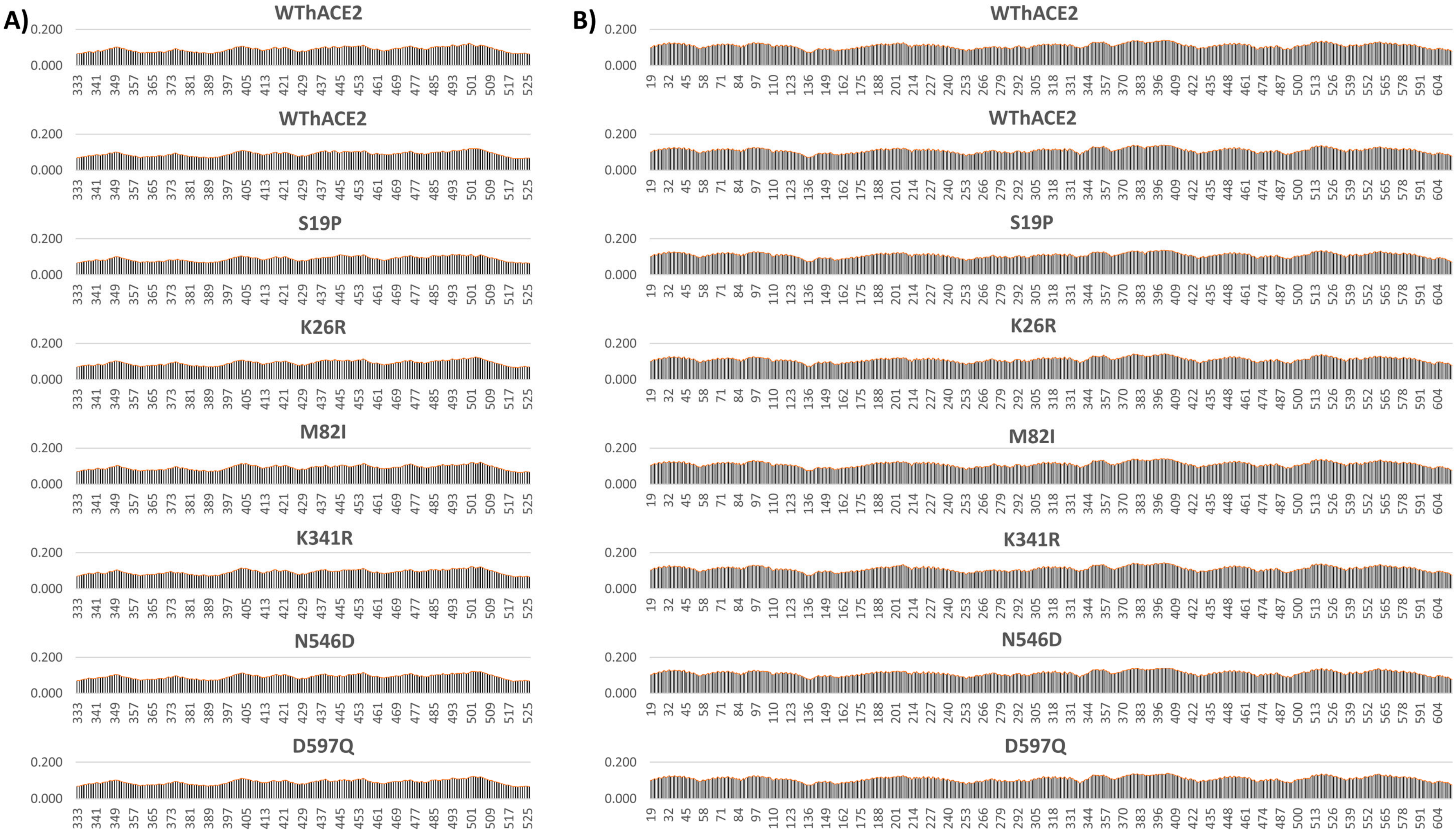

Figure S6: A) and B) show the *CC* distribution in the RBD and hACE2 proteins for each system. The x and y-axes show the residue numbers and the centrality values, respectively.

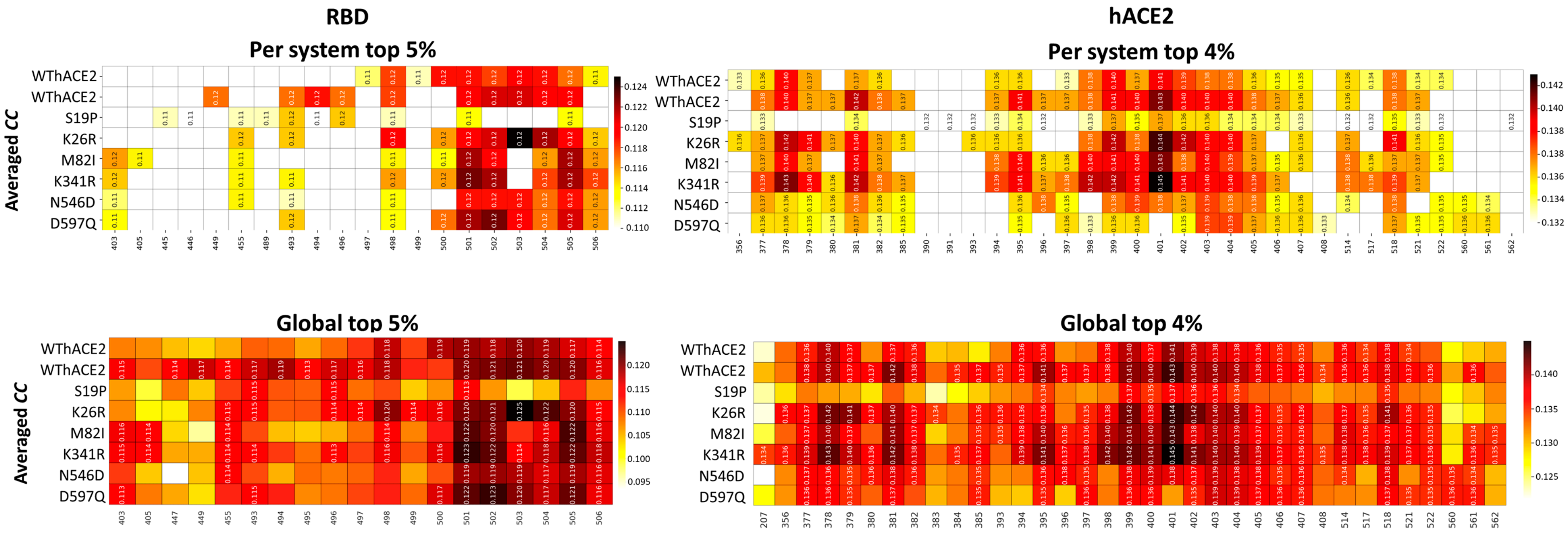

Figure S7: Heat maps of the top 5% and 4% *CC* high centrality residues in the RBD and hACE2, respectively, at an individual protein level and global level. Resides are on the x-axis and systems on the y-axis. The color scale from white to dark red shows the degree of centrality. The centrality color scale different for the local and global residues because in the case of local analysis, centrality calculation is based on the individual systems as opposed to the whole ensemble under global analysis.

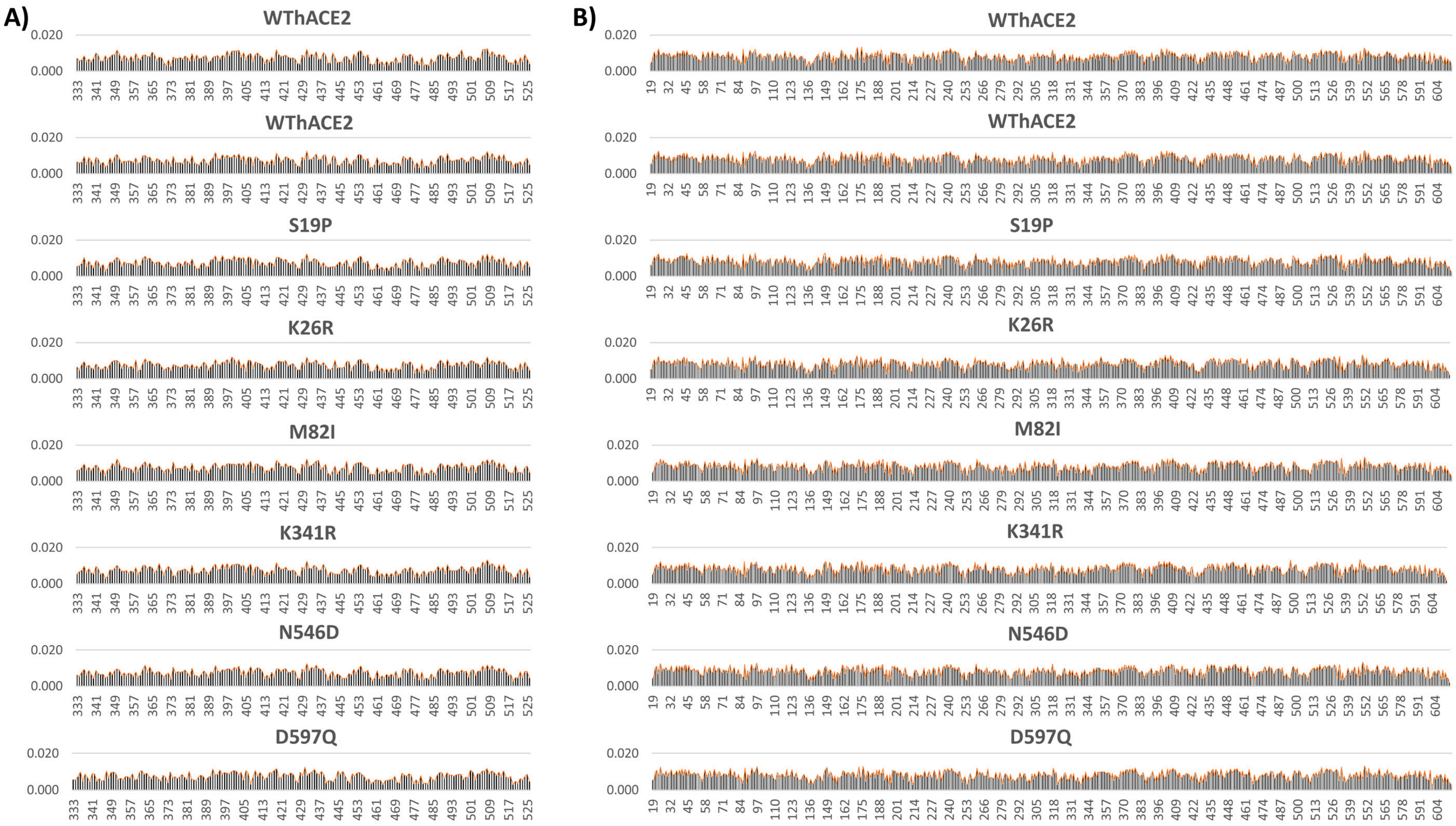

Figure S8: A) and B) show the *DC* distribution in the RBD and hACE2 proteins for each system. The x and y-axes show the residue numbers and the centrality values, respectively.

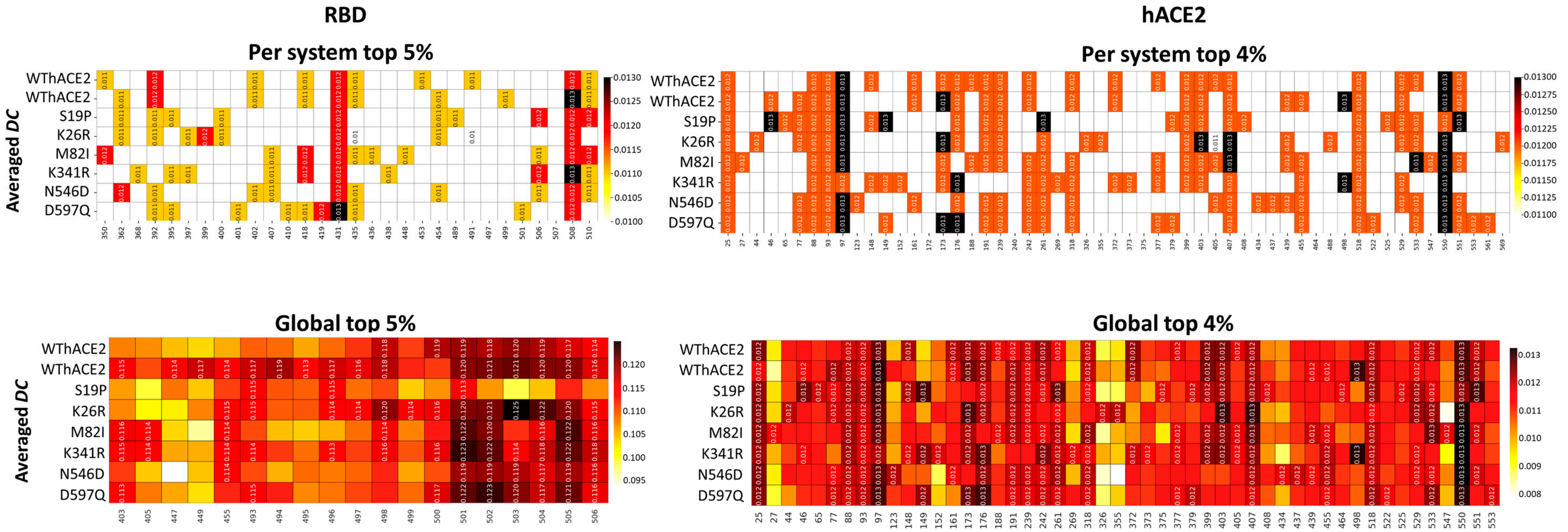

Figure S9: Heat maps of the top 5% and 4% *DC* high centrality residues in the RBD and hACE2, respectively, both at the individual protein level and global level. Resides are on the x-axis and systems on the y-axis. The color scale from white to dark red shows the degree of centrality. The centrality color scale different for the local and global residues because in the case of local analysis, centrality calculation is based on the individual systems as opposed to the whole ensemble under global analysis.

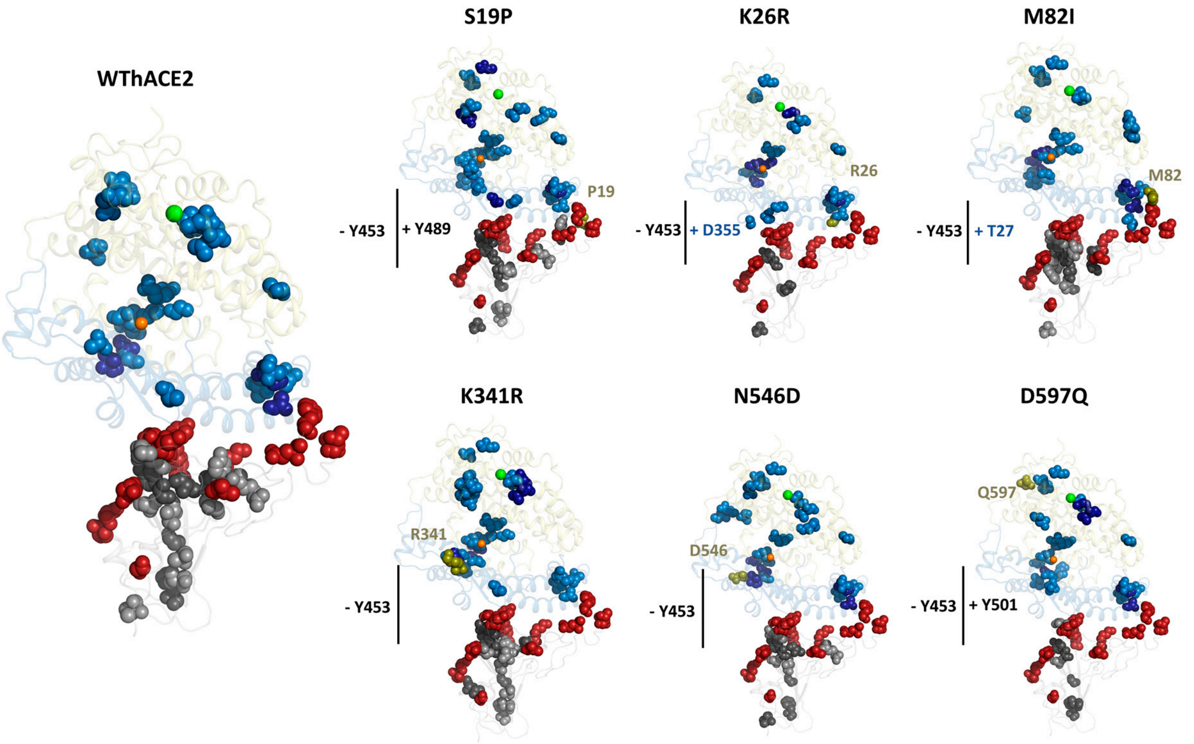

Figure S10: The distribution of top 5% and 4% RBD and hACE2 residue *DC* hubs in the RBD-hACE2 complexes. For each system, RBD is shown as a grey cartoon and hACE2 sub-domains I and II as sky-blue and pale-yellow, respectively. Zinc and chloride ions are shown as orange and green spheres, respectively, whereas the hACE2 mutations as deep olive spheres. System hubs are shown as sky-blue spheres (hACE2) and grey spheres (RBD). The five highest centrality *DC* hubs in the RBD and the hACE2 are shown as dark grey and dark blue spheres, respectively. The gains and losses in interface residue hubs in the hACE2-mutation containing systems compared to the WThACE2 system are indicated with – and +, respectively. The hACE2 mutations are shown as raspberry spheres.

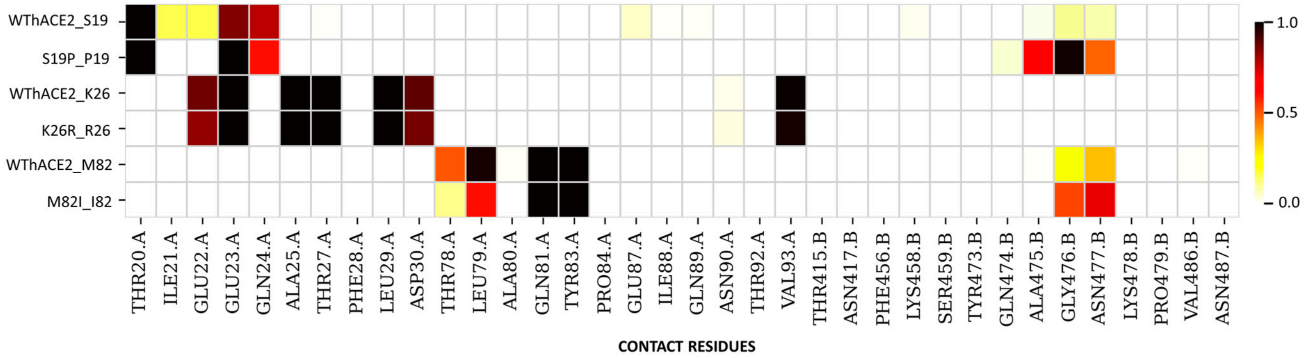

Figure S11: Heat map of the interface hACE2 variation residue contacts for the WThACE2 and hACE2 variant systems. The color scale from white through yellow to dark red indicates the degree of residue contact with dark being 1 and white being 0.
